# Boosting the mobility of ions in nanoporous confinement by applying a gate voltage

**DOI:** 10.1039/d6sc03773a

**Published:** 2026-10-01

**Authors:** Peng Qin, Modan Liu, Abhinav Chandresh, Yunzhe Jiang, Lars Heinke

**Affiliations:** a Karlsruhe Institute of Technology (KIT), Institute of Functional Interfaces (IFG) Hermann-von-Helmholtz-Platz 1 76344 Eggenstein-Leopoldshafen Germany Modan.Liu_at_KIT.edu; b Freie Universität Berlin, Institute of Chemistry and Biochemistry Arnimallee 22 14195 Berlin Germany Lars.Heinke@FU-Berlin.de

## Abstract

The mobility of ions under confinement in nanoporous host materials is central to many electrochemical energy transformation and storage devices. By using ionic liquids (ILs) as electrolytes and metal–organic frameworks (MOFs) as well-defined, regular nanoporous hosts, the dynamic ion properties were explored in detail. Here, we demonstrate for the first time that applying a gate-voltage (perpendicular to the charge transfer direction) allows the ion mobilities to be considerably enhanced. The experiments, which are based on MOF films, show a conductivity increase of up to 35%. In addition, molecular dynamics simulations provide deeper insights, showing that the gate-voltage-field causes layers of increased and decreased ion concentrations, parallel to the charge transfer direction. In these layers, the ion mobilities is up to 2.5 times higher than that in the bulk phase or without an additional gate-voltage field. Thus, these layers of highly mobile ions could be referred to as gate-voltage-induced “ion highways”, causing the experimentally observed higher conductance. Moreover, a general correlation between the ion mobilities and the local ion concentration was found. The study provides deeper insights into ion dynamics in nanoporous confinement and provides a method for enhanced ion mobilities.

## Abstract 2

The mobility of ions confined in nanoporous MOFs is enhanced by applying a gate voltage. Simulations reveal “gate-voltage-induced ion highways” with locally enhanced ion mobilities and provide insight into the ion transport under confinement.

## Graphical abstract

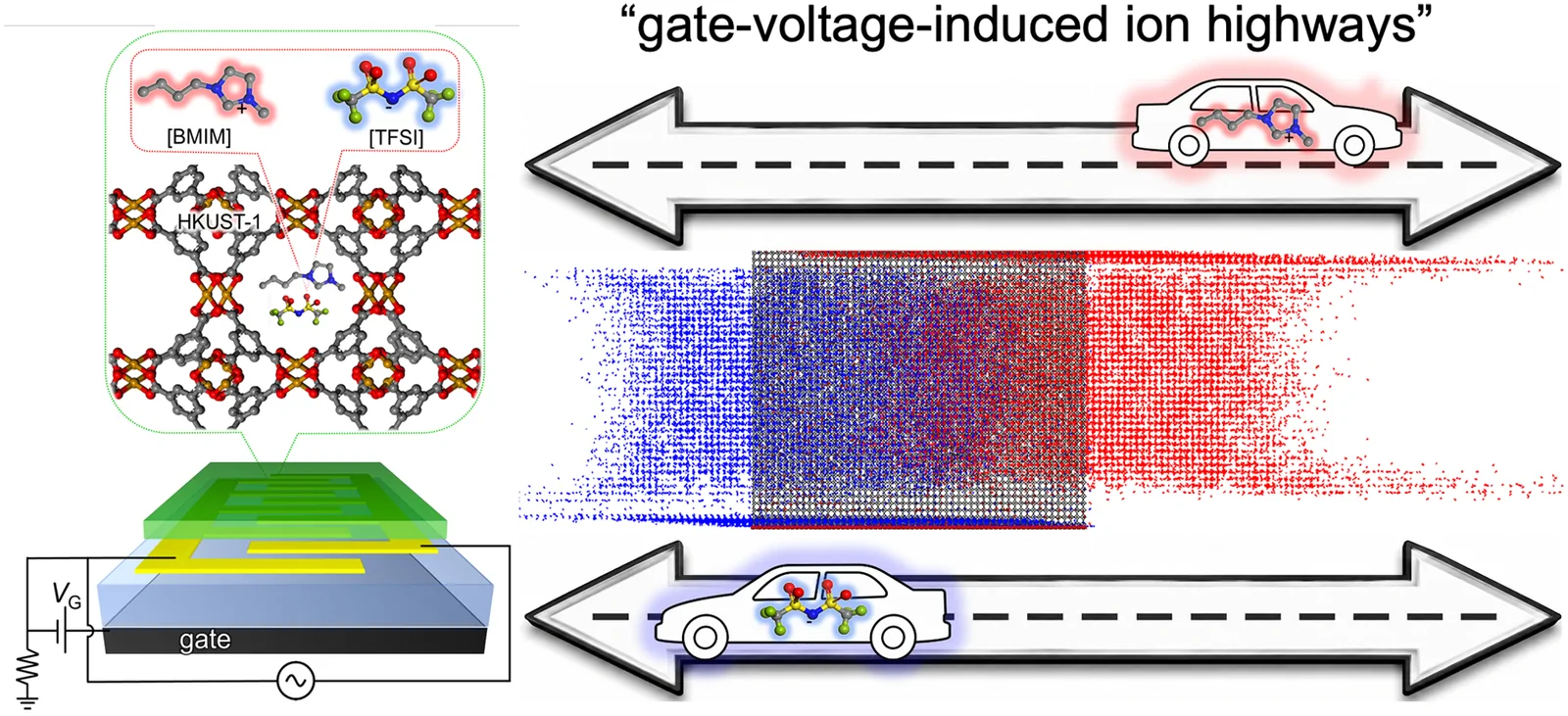

## Introduction

In many energy-storage-related applications, like in supercapacitors and batteries, the ions of the electrolyte are confined in a porous material, which is the electrode.^1–4^ For increasing the safety, the durability and the performance in many applications, a very promising path is to substitute conventional electrolytes with ionic liquids (ILs).^5–8^ ILs are organic salts with melting points below room temperature.^9–11^ Most ILs show exclusive properties, such as a negligible vapor pressure, non-flammability, an excellent thermal stability, a high ionic conductivity and a wide electrochemical window. Often, ILs are embedded in nanoporous materials, typically without further solvents. The physical and chemical properties of an IL, comprising the local structure, the melting point and the glass transition temperature, are severely affected by its confinement in the nanopores.^12–14^ In energy-storage-related applications like in supercapacitors, the mobility of ions confined in nanoporous materials is of utmost importance, determining the power-output and the charging rates. Unfortunately, ILs under confinement often show low mobilities, resulting in sluggish charging dynamics, which represents a severe problem for most applications.^5,8,15^ A better understanding of the structure–property relationship is needed to enhance the mobility.

Another important field of ions under nanoconfinement where the ion mobilities is of utmost importance is the field of ion-based information transfer (sometimes also referred to as “iontronics”).^16,17^ For example, in an asymmetric supercapacitor-diode (CAPode),^18–20^ the ions are confined in porous electrodes. Higher ion mobilities are aimed for, which allows faster switching and higher switching frequencies.

Metal–organic frameworks (MOFs) are a class of porous, crystalline materials with regular micropores.^21–23^ The MOF structures are controlled by the choice of the MOF components, which are linker molecules and metal nodes. Due to their unique properties, like the very high surface area, the well-defined regular pore structure and the good stability, MOFs possess great potential as host materials for ILs in composite electrolytes.^24^ It was found that ILs embedded in MOFs can enhance electrochemical properties, including an improvement in interfacial wettability and an increase in ionic conductivity in lithium batteries.^25,26^ A superior performance as supercapacitors was also found for conducting MOFs filled with ILs,^27^ where such organic ions form stable electric double layers and the MOF material is polarized.^28^ In these uses, the ion mobilities and the ionic conductivity of the IL electrolyte in the pores are key features. The ionic conductivity of the material is typically experimentally explored by electrochemical impedance spectroscopy (EIS), where the IL@MOF material is either in the form of a thin film on a solid substrate with deposited electrodes or in the form of powders, pressed into pellets between 2 planar electrodes.^25,29,30^ Previously, we used homogeneous MOF films on a solid substrate with deposited interdigitated electrodes, where the excess IL can be easily removed and only the conduction of the ions in the MOF pores is measured.^29–31^ For the IL of type 1-butyl-3-methylimidazolium bis(trifluoromethylsulfonyl)imide, referred to as [BMIM][TFSI], in the pores of the prototype MOF-structure HKUST-1,^32^ a mutual pore blockage was found at high IL loadings when an electric field (*E*-field) is applied. It is caused by cations and anions reciprocally blocking the pore windows when they drift in opposite directions through the pore system. This pore blockage causes the ions to accumulate at the blocked pores and their mobility drops tremendously.^31^ The conduction drop by a few orders of magnitude is generally unwanted in applications. A similar conduction drop, caused by the cations and anions mutually blocking the pores, connected with ion bunching, was also found in UiO-type MOFs.^33^

For proton-conducting electrolytes, like aqueous KCl solution, in mesoporous silica, it was demonstrated that the conduction can be controlled by applying a gate voltage perpendicular to the conduction direction.^34^ The gate-voltage induced an increase and a decrease in the conduction, which was explained by an increase or decrease of the cation and anion concentration in the pores as a result of the applied potential. Similar gate-voltage controlled proton-conduction was also found in other protonic materials, allowing the fabrication of protonic field-effect transistors (FETs).^35–39^ For these protonic materials, the applied gate voltage has a severe impact on the charge carrier concentrations. This is in contrast to ILs as electrolytes where the total numbers of cations and anions are constant. The control of conductivity and mobility of ILs by using a gate voltage has not yet been reported.

Here, the mobility of ions in nanoporous confinement is explored when a gate voltage, perpendicular to the conduction direction, is applied. To this end, we use an IL of type [BMIM][TFSI] in pores of HKUST-1 MOFs as a prototype example, Fig. 1. The experimental data based on impedance spectroscopy show that the conductivity of the entire sample can be enhanced by up to 35% when a gate voltage is applied. A thorough theoretical analysis based on molecular dynamics simulations shows that the biased IL distribution induced by the gate voltage results in channels with local low ion density regions, which results in a strongly boosted ion mobilities. These layers of highly mobile ions, which we refer to as “gate-voltage-induced ion highways” (or “gate-voltage-induced fast ion channels”), lead to an overall increase in the conductivity of the combined IL@HKUST system.

**Fig. 1:**
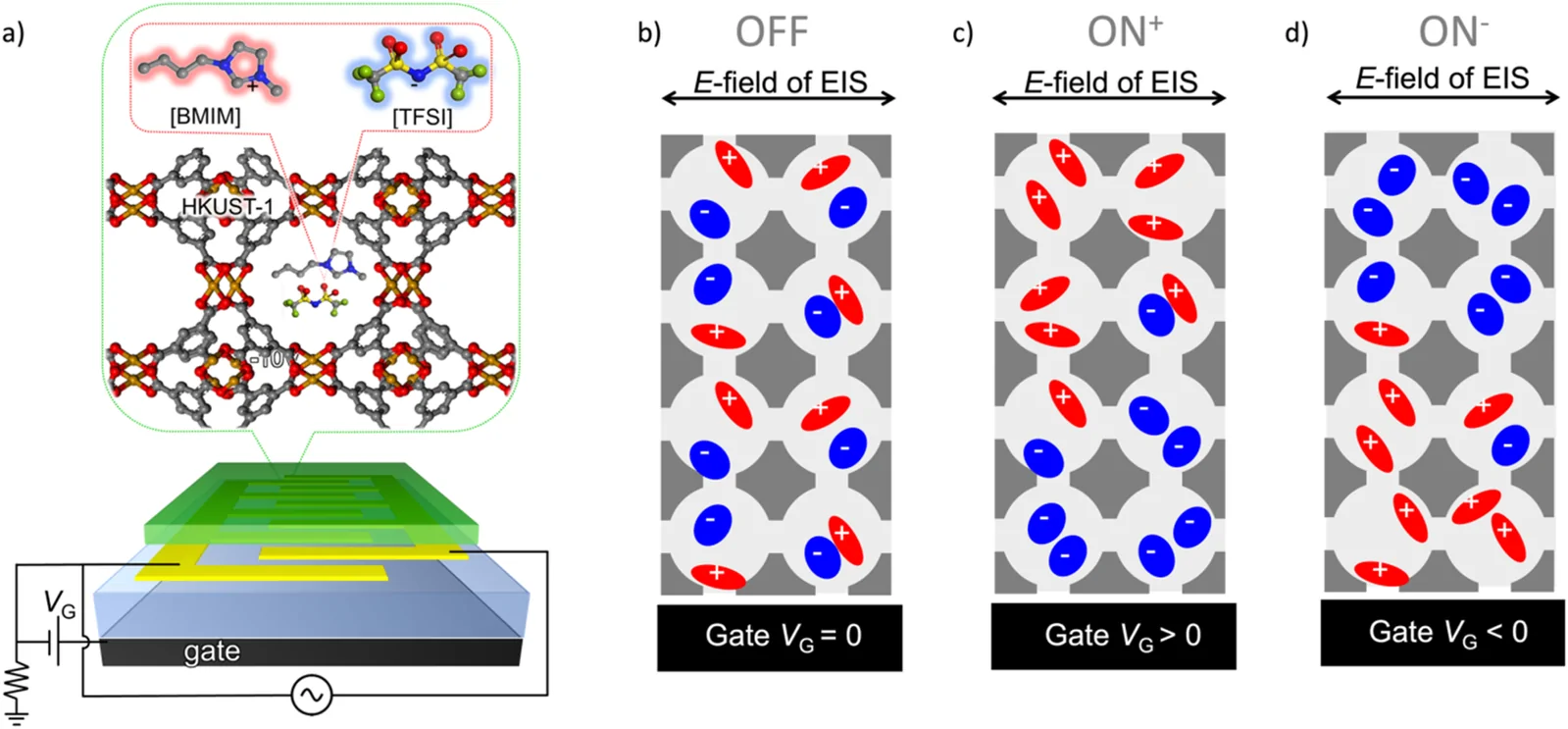
(a) IL of type [BMIM][TFSI] confined in the nanopores of HKUST-1 MOF. The color code for the atoms is C grey, O red, Cu orange, N blue, F green and S yellow, while H is not shown. Below: the MOF film (green) was grown on the substrate (light blue) with the deposited interdigitated gold electrodes (yellow). The ionic conduction was explored by electrochemical impedance spectroscopy (EIS), connecting the interdigitated electrodes. The substrate is silicon oxide (light blue) with a thickness of 230 nm on silicon (black) where the gate voltage is applied. (b) Sketch of the IL distribution in the pores. When no gate voltage is applied (OFF), the cations (red) and anions (blue) are essentially homogeneously distributed. (c) When a positive voltage is applied (ON^+^), the anions are attracted and the cations are repelled by the gate electrode, causing an anion enriched and a cation depleted region. (d) The opposite ion distribution is expected for a negative gate voltage (ON^−^).

## Results and discussion

### Measuring the gate-controlled ionic conductivity of IL@HKUST-1

The HKUST-1 MOF film was prepared directly on the substrate with the interdigitated electrodes, Fig. 1a. The films were made in a layer-by-layer fashion, also referred to as surface-mounted MOFs, SURMOFs.^40,41^ The crystallinity was explored by X-ray diffraction, Fig. S1, showing that the MOF film is crystalline and has the targeted HKUST-1 structure. The data also show the HKUST-1 is grown on the substrate with the crystalline [111] direction perpendicular to the substrate. Scanning electron microscopy (SEM, Fig. S2) images show that the MOF film has a uniform morphology, composed of many crystallites. Based on the cross-section images of the broken sample, the thickness of the MOF thin film was estimated to be 200 nm.

The loading of the pores of the MOF film with an IL of type [BMIM][TFSI] was performed by immersing the sample in IL–acetonitrile solutions, as previously optimized.^29–31^ Acetonitrile was chosen as solvent because of its mixability with [BMIM][TFSI], ensuring a homogeneous dispersion of the IL in the MOF, and its high volatility and rapid evaporation from the MOF pores, leaving only the IL in the pores.^30^ Moreover, the low conductivity of acetonitrile (and of the HKUST-1 MOF host) ensures that the measured conductivity can be attributed to the IL only. Following these previous studies,^29–31^ the MOF pores were loaded with 20%, 50% and 100% IL. The percentage refers to the IL filling of the free, accessible pore volume of the MOF film.

The ionic conductivity of IL@MOF samples was explored by electrochemical impedance spectroscopy (EIS). In addition to the 2 contacts at the interdigitated gate electrodes, allowing the ionic conduction of the IL@MOF film to be measured, an additional voltage was applied at the gate contact. The measurements were performed with the same sample and successive loadings of different IL percentages. Fig. 2 shows the Nyquist plots of the EIS data of the sample with different pore fillings at 0 V and ±100 V gate voltages. Further data, *i.e.* Nyquist plots for gate voltages of ±20 V, ±40 V, ±60 V and ±80 V, are shown in Fig. S3–S5.

**Fig. 2:**
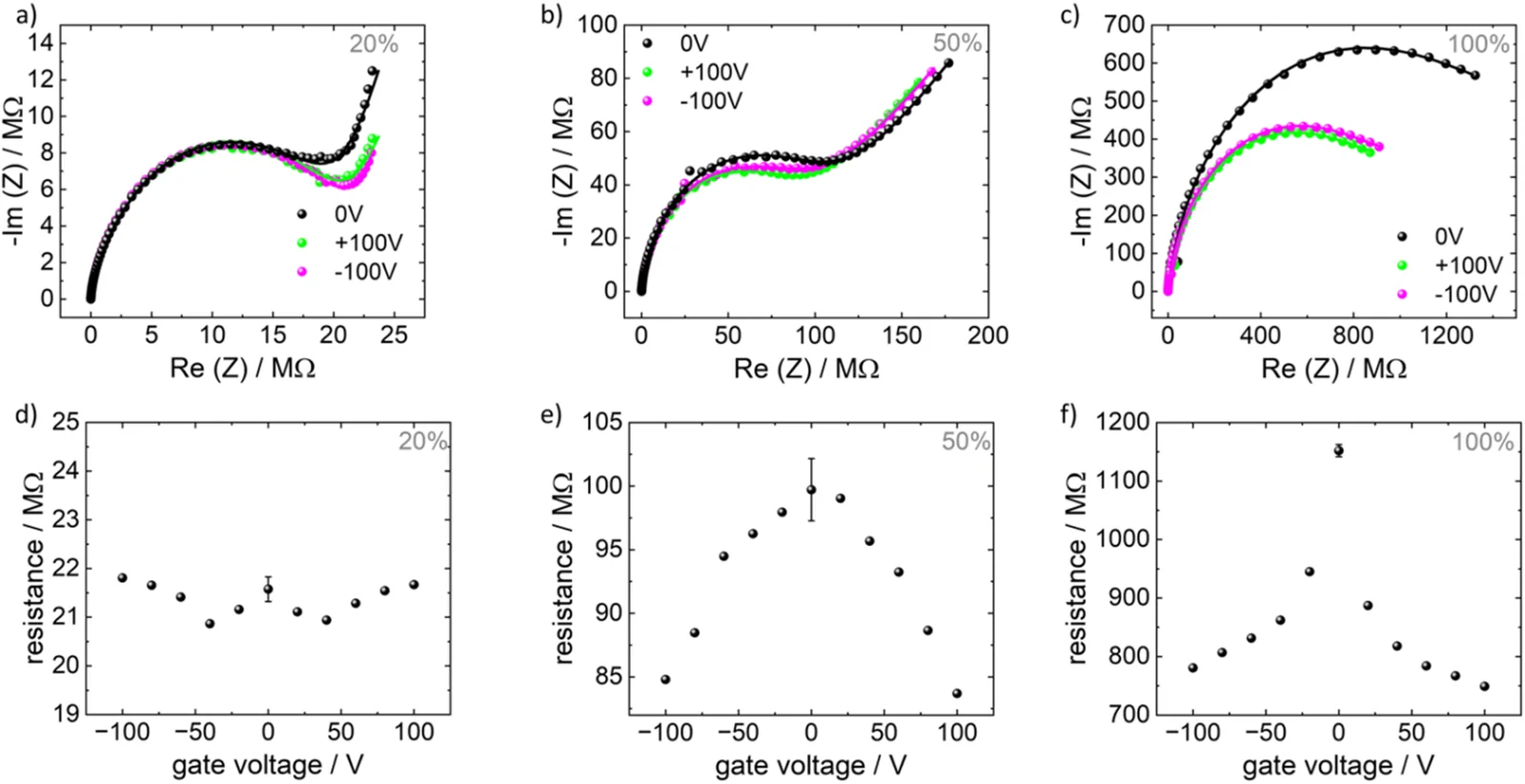
(a–c) Nyquist plots of the impedance data of the IL@MOF film with different pore fillings (a) 20%, (b) 50% and (c) 100% and various gate voltages, *i.e.* 0 V (black), +100 V (green) and −100 V (magenta), see labels. More Nyquist plots with different gate voltages are given in Fig. S3–S5. The frequency ranges of the data are 5 MHz to 0.5 Hz (a and b) and 5 MHz to 0.1 Hz (c). The colored spheres represent the experimental data and the lines are the fits with the (*R*–CPE_dl_)‖CPE_geo_ equivalent circuit,^42^ see also Fig. S3. (d–f) The determined resistance values *versus* gate voltage for IL pore fillings of (d) 20%, (e) 50% and (f) 100%. The error bars (at 0 V) are the standard deviations of the repeated measurements. All determined parameters are given in Table S1.

For analyzing the impedance data and to determine the ionic conductivity, an appropriate equivalent circuit of type (*R*–CPE_dl_)‖CPE_geo_, see ref. 42 and see Fig. S3a, was used. By fitting the experimental data with the equivalent circuit model, the ohmic resistance *R*, which is the ohmic resistance of the IL electrolyte in the HKUST-1 pores, was determined. The ohmic resistances of the sample (with different IL-filling levels) as a function of the applied gate voltages are shown in Fig. 2d–f. For the high pore loadings (100%, but also 50%), the resistance clearly decreases with increasing gate voltage. Similar resistance changes are obtained for negative and positive gate voltages.

With the ohmic resistance, the film thickness of the sample (see Fig. S2) and the geometry of the electrode (see the Experimental section), the conductivity can be calculated. The determined conductivity of the sample, Fig. 3a, is in a range of 1–60 µS m^−1^ (at 0 V gate voltage), which is much higher than the conductivity of the empty HKUST-1.^43^ Thus, the determined conductivity can be solely attributed to the IL electrolyte. The conductivity of [BMIM][TFSI] showed a dependence on the pore filling. Specifically, increasing the pore filling from 20% to 100% decreases the conductivities from 58 to 1.1 µS m^−1^. Such a strong decrease in the IL conductivity is in agreement with previous work, where the ions mutually block the MOF pore windows.^31^

**Fig. 3:**
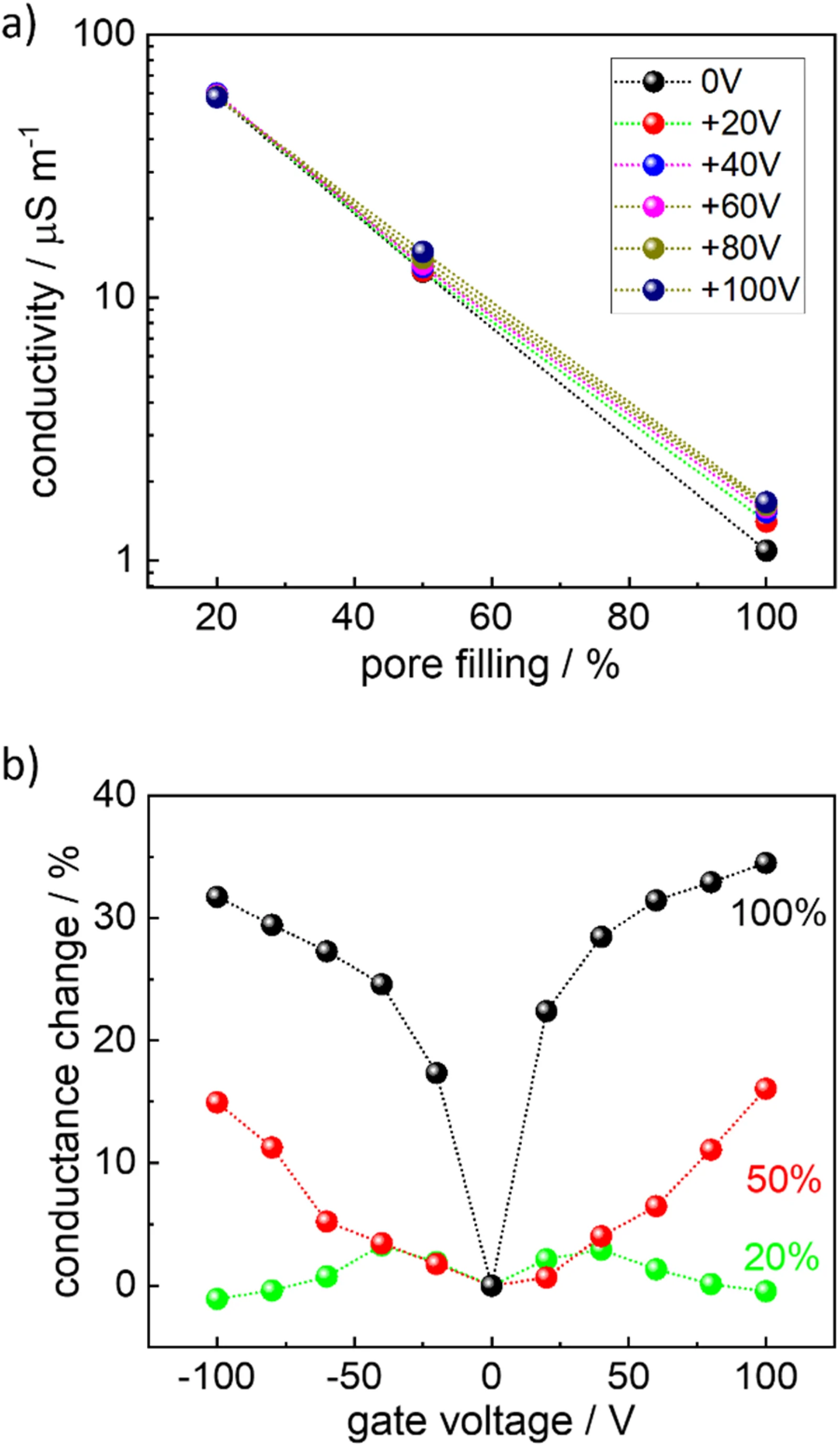
(a) Conductivity of the [BMIM][TFSI]@HKUST-1 film as a function of IL pore filling. The gate voltage varies between 0 V and +100 V, see the legend. (b) Conductance change as a function of the gate voltage. The IL pore filling is 20% (green), 50% (red) and 100% (black). The reference conductance values are obtained at a gate voltage of 0 V.

Fig. 3b shows the changes in the conductivity of [BMIM][TFSI] *versus* the applied gate voltage, with the 0 V-gatevoltage values as references (magnifications are shown in Fig. S12.) The data clearly show that, for large IL pore loadings, the IL conductivity increases with increasing gate voltage. The data also show that the effect of the gate voltage is significantly larger for the 100%-loading, compared to the smaller loadings. In detail, at 100% loading of IL in the MOF pores, a gate voltage of +100 V or −100 V increases the conductivity by ≈35%. On the other hand, for the sample with only 50%-IL-loading, a gate voltage of +100 V or −100 V increases the conductivity by ≈16%. For small loadings, the conductivity is roughly constant. A detailed inspection shows a slight conductivity maximum at ±40 V (by ≈3% more than that at 0 V-gate-voltage), where the conductivity slightly decreases again towards larger gate voltages. These changes are negligibly small compared to the conduction changes in the sample with larger IL loadings.

Generally, when a gate voltage is applied to the bottom of the substrate (see Fig. 1), the gate electrode attracts the counter ions, which move through the MOF pores to the bottom of the MOF material. This results in the separation of cations and anions in the material, creating cation-enriched and anion-enriched regions, as sketched in Fig. 1c and d. For example, by applying a positive voltage to the gate electrode, anions are attracted and enriched in the bottom area of the MOF film. Meanwhile, cations are repelled from the gate, forming a cation-enriched area in the top region. As a hypothesis, this separation phenomenon with locally unbalanced cation–anion ratios attenuates the mutual pore blockage of the cations and anions in the pores (which results in small ion mobilities) and, thus, enhances the ionic conduction. To gain a deeper understanding, we performed molecular dynamics simulations.

### Coarse-grained molecular dynamics simulations for the gate-controlled ion mobilities

To study the IL transport along the direction of the electric field (here the *x*-direction in the pore channels) as well as the IL distribution at the perpendicular gate voltage (here in the *z*-direction), we have developed a coarse-grained (CG) model for molecular dynamics (MD) simulations. Compared to the all-atom representation in the previous studies,^29,31^ the CG scheme allows for the investigation of dynamic properties on much larger length and time scales. This is crucial in revealing the ion distribution and double layer formation caused by the gate voltage. In the previous studies, the *E*-field drives the cations and anions in opposite directions. Depending on the IL loading, the limited MOF pore aperture inflicts a critical transition from homogeneous ion flows for low IL loading over transient stop-and-go for medium IL loading to terminal failure of IL transport for high loadings. This is caused by the pore windows which are mutually blocked by the cations and anions when simultaneously drifting in opposite directions. This results in the formation of IL inhomogeneities with layers of highly concentrated, bunched ions (for more details, please see ref. 29, 31 and 33.)

During the transition from mobile to immobile IL, the IL size *versus* the MOF pore aperture is the critical parameter. In particular, the [TFSI] anion with a rather rigid molecular structure is effectively larger than the [BMIM] cation with a rigid aromatic “head” and a flexible aliphatic tail. To reflect the dynamic properties for both, our CG representation utilizes 3-site CG representations for [BMIM] and [TFSI], Fig. 4a. On the MOF side, HKUST-1 features a unique large-cavity/small-opening scheme with pore apertures of 0.7 nm in diameter and pore bodies of 1.3 nm in diameter. (Ignoring the van der Waals radius, the diameters are 0.9 and 1.5 nm as atom nucleus-to-nucleus distance. The secondary pores with an aperture < 0.4 nm are inaccessible for individual [BMIM] or [TFSI]). In CG modelling, Lennard-Jones interactions define steric size exclusion in dimensionless CG units, where parameters such as mass, charge and bond length scale proportionally to the all-atom representation. However, establishing the temporal scaling factor between the coarse-grained and the atomistic model, *τ*_CG_, requires additional correlation between CGMD simulations and experimental data. In this work, arbitrary units are used for the ion mobilities and ionic conductivity. The details of the parametrization for the CG model are described in the Methods section.

**Fig. 4:**
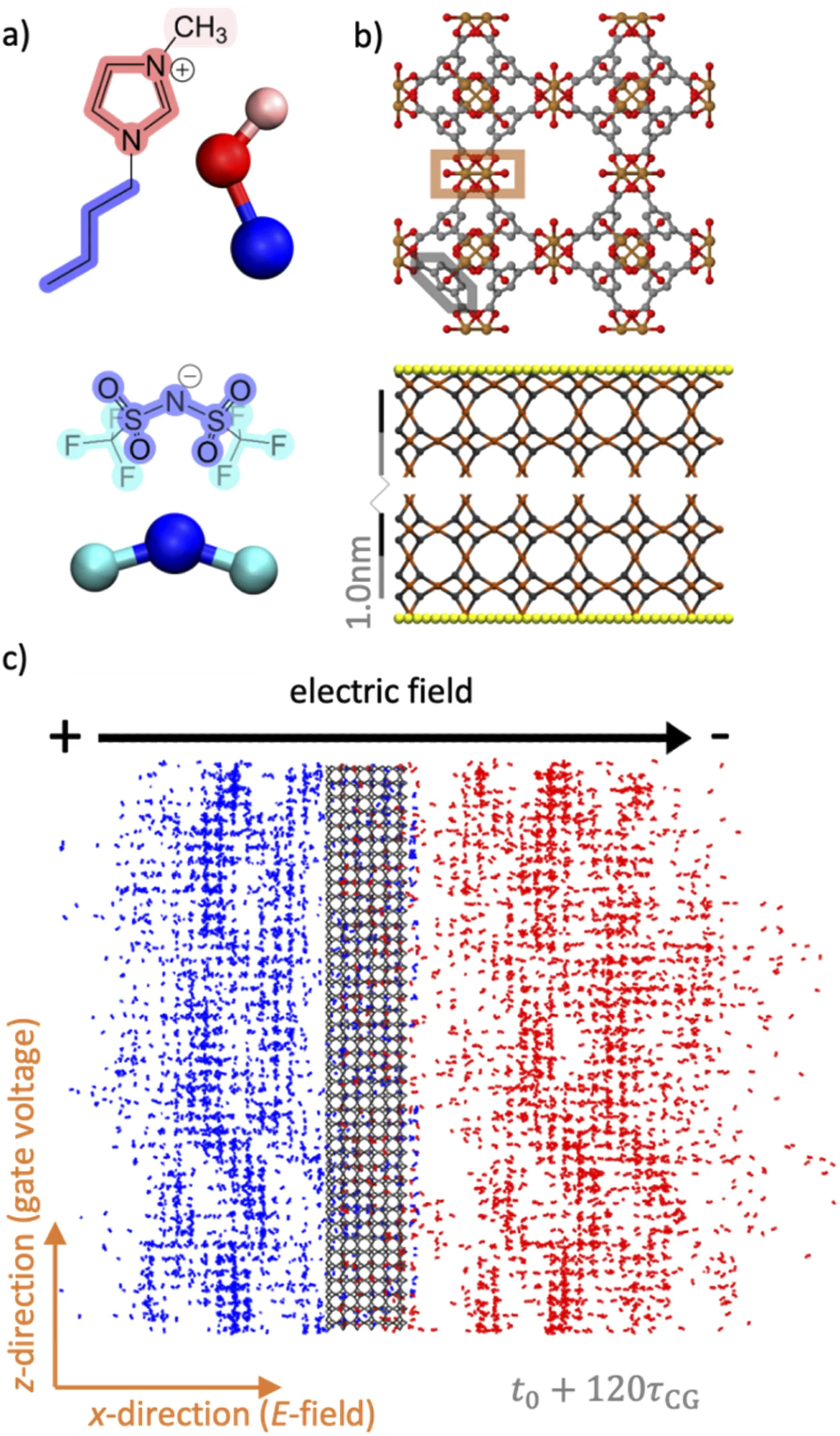
(a) Coarse-grained (CG) representations for individual [BMIM]^+^ and [TFSI]^−^ ions. (b) Top: all-atom representation of the HKUST-1 unit cell. Bottom: a CG supercell with an explicit CG electrode. The CG HKUST-1 consists of SBUs (brown) and linkers (gray). The electrode is presented by layers of gold atoms, shown as yellow spheres. Scale bar segments (grey) represent 0.5 nm per block. For the MOF–electrode interfaces, linker–electrode and SBU–electrode contacts correspond to minimal and small ion channels whose cavity is comparable to the size of half the MOF channel. (c) Initial phase in the formation of layers with inhomogeneous ion distributions of [BMIM][TFSI]@HKUST-1 with 100% pore filling, reproduced in the CGMD simulation (without gate electrodes). Initially, the cations (red) and anions (blue) are homogeneously embedded in the MOF (grey). After onset of the external electric field (at *t*_0_), the cations and anions drift in opposite directions along the *x*-axis, shown by the “unwrapped” coordinates. Cations drift to the right and anions drift to the left. In the snapshot at *t*_0_ + 120*τ*_CG_, layers of IL concentration inhomogeneities gradually form as the IL mobility is bottlenecked by the MOF pore aperture, in conformation with the all-atom simulation from previous studies.^31^ Periodic images of the MOF host are omitted to improve clarity.

The gate electrodes in CGMD are placed in direct contact with the MOF to prevent gaps between the electrode and the MOF. Even without application of a gate voltage, a limited number of ions is immobilized within the MOF–electrode interface due to the small cavity. In the remainder of the system, the ion distribution remains homogeneous along the *z*-direction. When a gate voltage is applied, the cations drift towards the negative gate electrode and accumulate there, while the anions drift towards the positive gate electrode and accumulate there. This induces an inhomogeneous IL distribution along the *z*-direction, as illustrated in Fig. 5a. In the experiment, the gate voltage is applied to a single-plate electrode, embedded in the substrate. In the simulation, a double-plate electrode setup is utilized. As ions are driven both in the *x*-direction (with periodic boundary conditions in, both, *x* and *y* directions) and in the *z*-direction with explicit electrodes, effectively blocking the IL, some ions are immobilized within the double layers at the MOF–electrode interface. It has to be noted that in the MD simulations, atomistic or coarse-grained, the driving force (*i.e.* the *E*-field) exerted on individual ions is set to be much higher than that in the experimental setup, to prevent kinetic trapping. The gate-voltage field is comparable to or larger than that in the *x*-direction field, so the gate electric field induces a large inhomogeneity in the ion distribution, particularly for low IL loading. Here, we focus on the system with 100%-IL-pore filling, while the CGMD results of reduced pore fillings are discussed in the SI.

**Fig. 5:**
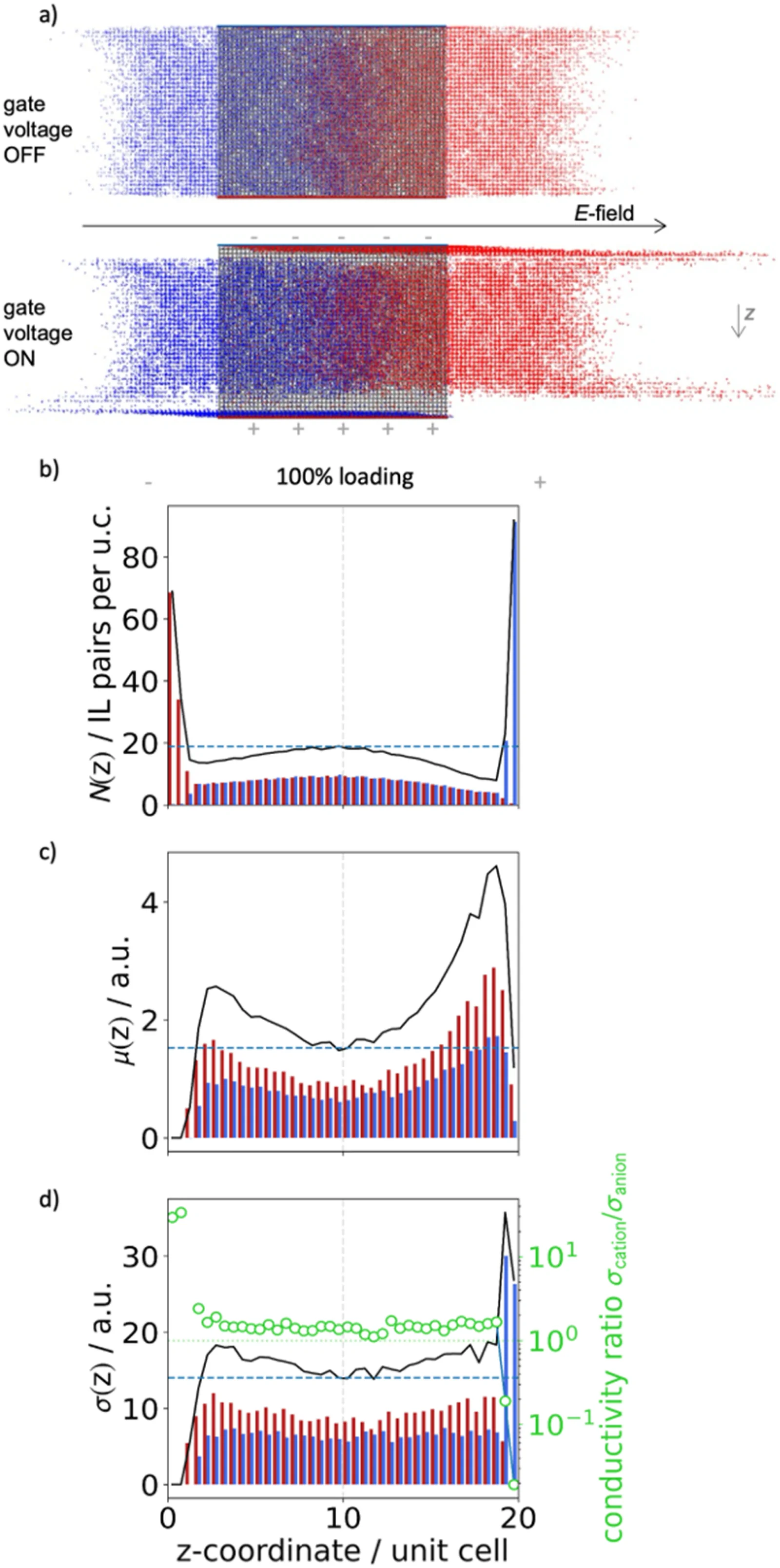
(a) IL@HKUST-1 snapshots of the simulations, without (above) and with (below) applied gate voltage. The electric field is along the *x*-direction, from left to right (see the arrow). The cations are shown in red and the anions are shown in blue. When no gate voltage is applied, the snapshot indicates homogeneous IL distribution along the *z*-direction. In contrast, when a gate voltage is applied, the cations and anions form layers at the positive and negative gate electrodes, respectively, with decreased and increased ion concentrations. The snapshots are obtained at *t*_0_ + 450*τ*_CG_. The loading of IL in the MOF pores is 100%. Distribution of the (b) ion density *N*(*z*) and (c) mobility *µ*(*z*) and (d) ionic conductivity *σ*(*z*) = *N*(*z*)·*µ*(*z*) *versus z*, for 100%-IL-loading. The average numbers from the bulk system (without gate electrodes) are shown by the blue dotted lines. The black lines are the common contribution from the cations and anions. The grey “+” and “−” indicate the positive and negative gate electrodes. The green spheres in (d) show the ratio of the conductivity of the cations *versus* anions, where the green dotted line represents a value of 1.

The density of ions with respect to the *z*-coordinate, referred to as *N*(*z*), is shown in Fig. 5b and S8. At a small gate voltage, the electric field is immediately screened in the *z*-direction using the ion layer formed at the MOF–electrode interface. The overall density of the IL in the center of the system (*i.e.* far away from the double layer) remains homogeneous with evenly mixed cations and anions like in the “bulk phase” (without gate electrodes). Here, the MOF–electrode interfacial double layers are able to hold a limited number of ions with a significantly larger IL density than in the “bulk” phase.

In addition to the counter-ions which are attracted by the gate electrode forming the electric double layer at the MOF–electrode interface, the co-ions are repelled by the gate field. This results in layers of low ion density, clearly visible as the minima of the ion density in Fig. 5b. These low-density regions are two-dimensional layers at both gate electrodes. The ion density in the center part of the system (at *z* ≈ 7–13) is similar to the density in the bulk system. A detailed inspection shows only a slight decrease. Compared to the ion density in the center part of the system, the density in the low-ion-density regions is much smaller. The IL density decreases by about 25–50%. In a wide range of these low-density regions, the cation–anion ratio is ≈1, like in the center of the system. In contrast, the regions close to the gate electrodes are dominated by the counter-ions and essentially no co-ions are present. This means that, at the positive gate electrode, all ions are anions and *vice versa*. Directly at the gate electrodes, the ions form a highly packed, pure cation or anion double layer. The density of this layer, which is only one-two unit cell wide (*i.e.* the thickness of the high-density ion region is approximately 1.5 unit cells, increasing to approximately 2 unit cell lengths with increasing gate voltage), reaches up to 60–80 ions per unit cell, which is considerably more than that in the bulk phase.

The mobility of the ions is shown in Fig. 5c. Most interestingly, the mobility in the low-density-regions is considerably enhanced. The ion mobilities in the low-density-layers of the 100%-IL-loading-system is boosted by a factor of ≈2.5. As expected, due to their similar ion density, which is only slightly decreased when compared to the zero-gate-voltage case (or the bulk system), the ion mobilities in the center part of the system is similar to the mobility in the bulk system, only marginally enhanced by ≈5%. In the highly packed layers directly at the gate electrodes, the ions are immobilized and the mobilities are essentially zero.

The conductivities, corresponding to the product of the ion mobilities and density, *versus z* are shown in Fig. 5d. The conductivity is enhanced over the major part of the system. In the low-ion-density-layers, the conductivity is increased by up to a factor of 1.3 and 2.5, respectively. The 2.5-times-conductivity-increase is realized for the layer at the positive gate electrode, where anions outnumber the cations. Although the anion mobility is not the highest in the system, the high anion density results in very large conductivities. Despite some regions also show a decreased conductivity by applying a gate voltage, the overall conductivity is enhanced.

For small IL loadings (Fig. S8a and b), the ion motion is rather fast and a mutual pore blockage of the ions, causing a significant decline of the conductivity, cannot be observed. This is in agreement with previous fully atomistic studies.^29,31,33^ As a result, the ion mobilities is not significantly enhanced by applying a gate voltage, as also found in the experiments (Fig. 3, 20%). A detailed inspection of the simulation data (Fig. S8a and b) shows that, when a gate voltage is applied, a significant amount of ions is immobilized at the electrode, which leaves fewer ions in the channels. On the other hand, the mobility of the mobile ions is enhanced (due to the decreased ion density). As a result of both opposing effects, the gate voltage causes a small, but non-monotonic response in the conductivity, see Table S3 and Fig. S8a.

Based on the data in Fig. 5d, the conductivity averaged over the entire system increases by approximately 10% when the gate voltage is applied, compared to the gate-voltage-free conductivity. This is in qualitative agreement with the experimental data, see Fig. 3b. More data for the simulated mobility and conductivity changes (for various loadings and gate voltages) are given in Tables S2 and S3.

For gaining deeper insights, we correlate the ion mobilities with the local ion density, Fig. 6. The plot shows that the ion mobilities decreases with increasing ion density. Remarkably, all data points from different average IL loadings fall on the identical master curve. Please note that the data without the gate field show a conduction drop by a factor of approximately 4, which is significantly smaller than the conduction drop observed in previous fully atomistic simulations.^29,31,33^ Nevertheless, the mechanism causing the conduction drop, *i.e.* the mutual pore blockage by the cations and anions drifting in opposite directions and causing ion accumulation, can also be observed in the CG simulations.

**Fig. 6:**
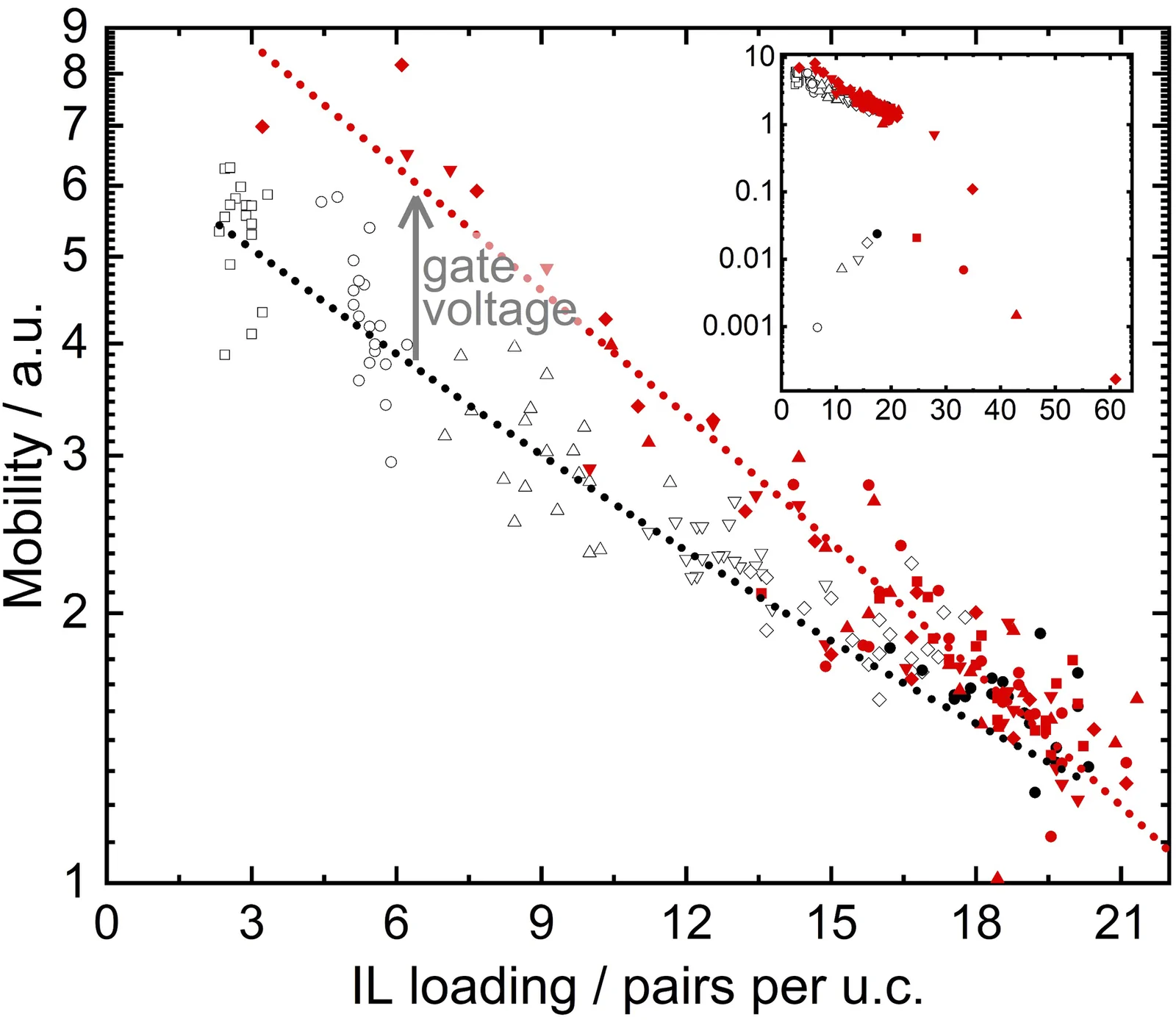
Correlation of the local IL mobility and the local IL density. The local density is averaged for each unit cell along the *z*-direction (see Fig. 5b–d). The red points are from the simulations with 100%-pore-loading and the gate voltage on. The gate voltages are 10 V, 20 V, 40 V, 80 V and 100 V for the red symbols with shapes of squares, circles, triangles up, triangles down and diamonds, respectively. The data when the gate voltage is off are shown in black, where the pore fillings are 100% for the solid circles, 85% for the open diamonds, 70% for the open triangles tip down, 50% for the open triangles tip up, 30% for the open circles and 15% for the open squares (the average pore fillings are 19, 16.2, 13.3, 9.5, 5.7 and 2.9 IL pairs per u.c.). The inset shows the plot with all data points, including the outlier points. The dotted lines are the trend line of the data (precisely, the lines are three-exponential functions fitted to the data).

For the gate-field-free data, the ion distribution along the *z*-axis is rather homogeneous and the data appear very close to the average loading (please note that the ion inhomogeneities as a result of the mutual cation–anion pore blocking are along the *x*-direction and averaged out here). In contrast, the ion distribution becomes inhomogeneous when a gate voltage is applied, see Fig. 6 (or S10). Even for full average loading, the ion density is widely distributed from very low to very high (as also shown in Fig. 5b). More importantly, the mobility is strongly enhanced. While the regions with the highest ion densities (*i.e.* the electric double layer at the MOF–electrode interface) have very low mobilities and thus do not contribute to the total conduction, the regions with low densities show a very high mobility. It is remarkable that, for the average 100%-loading, the ions in the regions with low local ion density show a significantly enhanced ion mobilities when compared to the gate-field-free data at similar densities. This means that the gate-field-induced layers of low ion densities are significantly more conducting than expected for this ion density. Thus, we refer to them as “gate-voltage-induced ion highways”. These highway layers are the low density/high mobility regions close to the positive and negative gate electrodes as seen in Fig. 5.

Due to the higher mobility of the cations compared to the anions, contribution of the cation-conductivity is approximately 50% larger than that of anion-conductivity when no gate field is applied. When a gate field is applied, the conductivity of the cation-rich and anion-rich layers is strongly dominated by the main charge carriers, see Fig. 5d. This means that close to the positive gate electrode, the conductivity of anions is about 30 times higher than that of the cations and *vice versa* at the negative gate electrode. This means that in the picture of the highway, these are lanes in opposite directions.

A detailed inspection of the CG simulation data shows that, first, the mutual pore blockage as observed for the gate-field-free system is strongly attenuated by the gate-voltage. This is primarily caused by the large number of ions which are immobilized at the interfacial double layer structure at the gate electrode and, thus, the IL density is reduced in the other regions. This reduction in ion density causes less frequent pore blockage, which mainly occurs at full IL loadings. Second, the ion distribution is off balance; this means that cation-rich and anion-rich layers are formed. In these layers the dominant ion-species are highly mobile, more mobile than those in the bulk layers of comparable density, see Fig. 6 and S10. The high mobility in these gate-voltage induced layers is caused by two effects: since most ions drift in the same direction, the mutual friction is reduced. In addition, since the co-ions repel each other, they do not form clusters and show a homogeneous ion distribution, resulting in a higher ion mobilities. Both effects further enhance the ionic conduction, compare red *vs.* black data in Fig. 6.

## Conclusions

The mobility of ions confined in nanoporous materials is enhanced by applying a gate voltage perpendicular to the charge transfer direction. We explore this effect by using an ionic liquid in the pores of a prototype MOF. We show experimentally that, depending on the pore filling, the gate field can enhance the ionic conductivity by up to 35%. By means of CGMD simulations, deeper insights were gained showing that the gate field results in an inhomogeneous ion distribution in the porous host material. While highly packed electric double layers are formed at the electrodes, layers with low ion densities but very high ion mobilities are formed. In these layers, the cation–anion ratio is off-balanced, further enhancing the ion mobilities. Thus, these layers of highly mobile ions are gate-voltage-induced “ion highways” with up to 2.5 times higher mobilities.

The study shows a novel path to enhance the ion mobilities in nanoporous confinement. In addition, it shows a general correlation between the ion density and mobility, independent of the average pore filling. We believe that, as shown for different FETs,^44,45^ decreasing the thickness of the gate-dielectric-layer will allow the device to be operated with much smaller gate voltages. Integrating this concept into practical supercapacitors or iontronic devices requires overcoming several challenges in (micro)manufacturing, such as the production of uniform MOF (or similar nanoporous materials) layers with reliable electrical contact and gate electrodes. Addressing these hurdles will be essential to translate the observed “ion highways” into real-world, high-performance applications. This study may help design advanced devices based on nanoconfined ions with improved dynamic properties.

## Experimental section

### MOF film synthesis and IL loading

The HKUST-1 MOF film samples were prepared in a layer-by-layer fashion, where the substrates were alternately exposed to the metal-node and linker solutions.^40,41^ MOF films with an HKUST-1 structure were prepared from ethanolic 1 mM copper acetate and ethanolic 0.2 mM trimesic acid (BTC) solutions. The back-gated field-effect transistor (FET) substrates were purchased from Sigma-Aldrich, article number FIPMS148. The thickness of the silicon oxide dielectric layer is 230 nm. For the used device, the total gap length and gap width of the interdigitated gold electrodes on the substrate were 10 mm and 2.5 µm, respectively. The FET substrate was treated with UV–ozone for 30 minutes to remove impurities and to support the SURMOF growth.

The loading of IL in the HKUST-1 MOF pores was performed by immersing the sample in a solution of IL/acetonitrile with varying IL concentrations (20%, 50% and 100%) using [BMIM][TFSI] as the IL. The amount of IL absorbed in the MOF pores is roughly proportional to the concentration of IL in the solution, as estimated in ref. 30 and 31. The immersion process was carried out at room temperature for approximately 20 minutes. After the immersion, the sample was rinsed briefly with acetonitrile for about 2 seconds (removing excess IL) and dried in a stream of pure nitrogen (evaporating acetonitrile). This process was explored and optimized in previous studies.^29–31^

### Sample characterization

A Bruker D8-Advance diffractometer was used to perform X-ray diffraction (XRD) analysis of the samples. The diffractometer is equipped with a LynxeEye detector and a copper anode X-ray source with a wavelength of *λ* = 0.154 nm.

SEM measurements were performed using a TESCAN VEGA3 thermionic scanning system. To avoid the charging effects, all samples were coated with a thin layer (3–4 nm) of platinum by sputtering under high vacuum.

### Measurement of ionic conduction properties

Impedance spectroscopy experiments were conducted using a Zurich Instruments MFIA impedance analyzer. The LabOne software was used to control the measurement process. Before the measurements, the device was stabilized for 2 hours. The measurement range of the device was chosen based on the sample being tested to ensure a high signal-to-noise ratio: a frequency range of 5 MHz to 0.5 Hz and to 0.1 Hz, respectively, was scanned with 100 logarithmically spaced points. The “high to >medium setup” was automatically selected and no further corrections were made to cable lengths or reference measurements. A Keithley SourceMeter Unit (SMU) 2450 was used to apply a gate voltage. All cables used in the setup were triaxial cables. An EPS150TRIAX probe station and a custom-made chamber purged with gas (here pure nitrogen) were used for the experiments. The entire setup was grounded to the grounding system of the power grid *via* 1 MΩ resistors. The impedance of the samples was tested at different gate voltages and the cycles of applied gate voltage were 0 V, +20 V, 0 V, −20 V, 0 V, +40 V, 0 V, −40 V, 0 V, +60 V, 0 V, −60 V, 0 V, +80 V, 0 V, −80 V, 0 V, +100 V, 0 V, −100 V and 0 V. The data were analyzed by fitting the experimental data to the (*R*–CPE_dl_)‖CPE_geo_ equivalent circuit.^42^ The leakage current (between the drain and gate) was always smaller than 1 nA. An example measurement is shown in Fig. S11.

### Molecular dynamics simulation protocols

The [BMIM][TFSI] transport in HKUST-1 is simulated by CGMD. HKUST-1 is represented with a “minimalistic model” by assigning one CG bead per linker/SBU from ref. 46. The force field parameters are converted to Lennard-Jones interactions by least-squares fitting from the original modified Buckingham parameters. In the IL@HKUST system, we consider the secondary pores in the MOF inaccessible to both the cation and the anion, as the van der Waals (vdW) radius for the cross-section of the ionic molecules at ∼0.41 nm is too large for the pore aperture ∼0.39 nm. In the previous work^31^ with all-atom simulation of [BMIM][TFSI] transport through HKUST-1 with the MOF modelled as a flexible framework, the IL ions only very seldomly entered the secondary pores along the diagonal axes. However the rate of cations or anions entering or exiting is too small compared to transport along the major pore channel in the *x*-direction; hence the contribution to the overall mobility is discarded in the CG representation. Additionally, dummy CG sites are artificially placed in the secondary MOF pores to block passage of IL ions. The CG HKUST-1 is considered rigid, when the dummy CG beads blocking the secondary pores remain fixed.

The CG model for [BMIM][TFSI] features 3-site representation for the cation and anion. For [BMIM], the parameters are taken from ref. 47, whereas the [TFSI] parameter is fitted to the density profile from all-atom simulations for IL@HKUST-1 at full loading and a zero external electric field in ref. 31, to match the first peak in the radial distribution function for [BMIM]–[TFSI] pairs at ∼0.47 nm for fully IL loading the CG representation. During the MD simulation of IL transport, the individual cation and anion molecules are considered rigid.

In general, by converting the all-atom model into the CG representation, the vdW interaction is down-scaled due to loss of details in the intramolecular and intermolecular interactions. In addition to the parameter fitting from the literature, we have rescaled the Lennard-Jones interactions by 120% for HKUST-1 and 112% for the IL, to reproduce the critical pore blockage at approximately 85% IL loading, in agreement with the fully atomistic MD simulations. Moreover, CG parameters are fine-tuned to capture the dynamic properties, *e.g.* the drifting velocity of [BMIM] along the *x*-direction should be 3 times faster than that of [TFSI] (as found in our previous fully atomistic simulations). In an IL@HKUST-1 CG model with periodic boundaries in all dimensions, the formation of ion-bunching layers with different IL concentrations can be observed; however the reduction of mobility *versus* IL loading is also down-scaled. For comparison, in the experiment, from low loading (20%) to full loading (100%) of IL in HKUST-1, the IL mobility decreased by 3 orders of magnitude. In the all-atom simulation with an exaggerated *x*-direction *E*-field, this low loading *versus* high loading mobility reduction is ≈2 orders of magnitude, while in the CG representation the effect is ≈0.5 orders of magnitude. The loss of internal degrees of freedom during coarse-graining thus corresponds to a rescaled time resolution of *τ*_CG_ in the simulations even if all other CG scales are set 1 : 1 to the real system (mass unit *m*_CG_ = 1 Da, length unit *λ*_CG_ = 0.1 nm, energy unit *ε*_CG_ = 1 kcal mol^−1^). The determination of *τ*_CG_ relative to the lab time is irrelevant in the current work as we consider exclusively the steady-state ion flows and relative changes in the IL mobility. Otherwise for dynamics, the determination of *τ*_CG_ would require time series data from both the experiment and CG simulations. All CG parameters are listed in Table S4.

For the CG model of IL@HKUST-1, ions are embedded in a supercell of 3 × 3 × 20 and 27 × 3 × 20. The maximum loading of IL is taken from the all-atom model. The full loading of IL is estimated to be 19 IL pairs per MOF pore, *via* dividing the accessible HKUST-1 pore volume^48^ (66%) by the vdW volume per IL pair calculated from ref. 49. In the *x*- and *y*-directions, the system has periodic boundary conditions, whereas in the *z*-direction, two monolayers of fcc CG sheets explicitly represent the gate electrodes. The explicit electrodes are placed adjacent to the MOF CG beads to block the IL transport across the *z*-boundaries. The electrode–MOF separation is set to minimal at 0.1 nm *z*-distance to prevent “leaking” or IL currents. Alternatively, for 0.5 nm *z*-displacement between the electrode and the MOF beads, an ion channel comparable to that of the bulk phase IL would allow for fast IL transport, dominating the overall IL mobility across the IL@HKUST-1 system, within the slit on the electrode–MOF interface akin to the “nanowetted interface” in ref. 29 and 50. During the simulation of IL transport, the CG beads representing the electrode are fixed.

The initial configuration is optimized by energy minimization using the conjugate gradient method, followed by relaxation with 0.6M *τ*_CG_ using the Nose–Hoover thermostat at 300 K. During the equilibration, the *x*-direction *E*-field is set to zero and the electrodes are set to zero potential. The production was run with 4.2M *τ*_CG_ using the Nose–Hoover thermostat and Nose–Hoover–Andersen barostat under ambient conditions of 1 atm and 300 K. In the production run, the *E*-field in the *x*-direction is set to 10 V nm^−1^, while for the electrodes, the constant potential method (ref. 51 and 52) maintains, in separate settings, ±0 V, ±10 V, ±20 V, ±40 V, ±60 V, ±80 V and ±100 V potential in the double-plate electrode setup. The gate voltage in the *z*-direction creates a bias in the IL distribution and induces a double layer. In particular, at the MOF–electrode interface, the cations are immobilized on the anode and anions are immobilized on the cathode in terms of *z*-direction movement, whilst the *x*-direction transport is still maintained. The CGMD simulations utilize a double-plate electrode setup, in contrast to the single electrode setup in the experiment. Please note that to simulate the electric field of the gate electrode, the simulation setup cannot comprise only one bottom electrode. To bridge this gap, during calculation of conductivity, the contribution is integrated from the MOF–electrode interface towards the center of the IL@MOF system, or the “bulk phase”. Since the two MOF–electrode interfaces are asymmetrical (SBU–electrode or linker–electrode), the integration from both interface types is utilized for the average values. In the simulation the MOF has 20 unit cells in the *z*-direction, and for the calculation of conductivity, *N*(*z*) and *µ*(*z*) are integrated over *z* ∈ (0,9) for the SBU–electrode interface and *z* ∈ (10,20) for the linker–electrode interface. Such integration towards the symmetry center provides an approximate representation of the experimental configuration while retaining the physically consistent electric field required by the simulations.

The mobility of the IL is determined by measuring the *x*-direction drift. Local IL density and mobility shown in Fig. 5 and 6 correspond to average velocity with a time interval of 100*τ*_CG_. The conductivity of IL is calculated as 
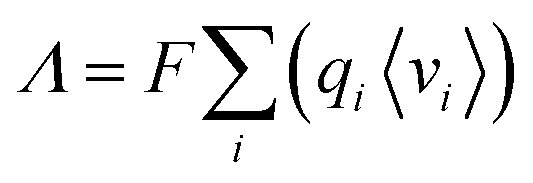
 by taking contributions from both the cation and anion. The *i*-th component corresponds to [BMIM] and [TFSI], *q* is the charge of the ion, *v* is the drift velocity in the *x*-direction and *F* is the Faraday constant. It is to be noted that the suppression of internal degrees of freedom results in physically oversized but soft coarse-grained representations of both the IL and the MOF. Although the relative cation and anion mobility ratio can be matched to the fully atomistic model, the reduced steric constraints systematically facilitate ion transport through densely packed regions. As a result, the mutual pore blockage (and, thus, the enhancement of conductivity through the gate voltage) can be weaker than that experimentally observed.

## Author contributions

PQ performed the experiments. AC and PQ analyzed the data. ML developed the CG model and performed the simulations. YJ recorded the SEM images. LH planned the project and the experiments. PQ, ML and LH wrote the manuscript, which was supported and approved by all authors.

## Conflicts of interest

The authors declare no conflict of interests.

## Supplementary Material

SC-OLF-D6SC03773A-s001

SC-OLF-D6SC03773A-s002

## Data Availability

Data for this study are available at the refubium repository at https://refubium.fu-berlin.de/handle/fub188/52393. Supplementary information (SI): PDF with additional data, tables and plots. See DOI: https://doi.org/10.1039/d6sc03773a.
