# Supplementary material for: Boosting the mobility of ions in nanoporous confinement by applying a gate voltage
Source: Chem Sci. 2026 Oct 1. Online ahead of print. doi: 10.1039/d6sc03773a (PMC13651806; doi:10.1039/d6sc03773a)
Supplement: Supplementary file 1 [file SC-OLF-D6SC03773A-s001.pdf]

Supplementary Information (SI) for Chemical Science.
This journal is © The Royal Society of Chemistry

# Supporting Information:
# Boosting the mobility of ions in nanoporous confinement by applying a gate voltage

*Peng Qin,[a] Modan Liu,[a] Abhinav Chandresh,[b] Yunzhe Jiang[a] and Lars Heinke [a,b]**

[a] Karlsruhe Institute of Technology (KIT), Institute of Functional Interfaces (IFG), Hermann-von-Helmholtz-Platz 1, 76344 Eggenstein-Leopoldshafen, Germany.
[b] Freie Universität Berlin, Institute of Chemistry and Biochemistry, Arnimallee 22, 14195 Berlin, Germany. Email: Lars.Heinke@FU-Berlin.de

**Table of Contents**

1. Additional Data
    - XRD
    - SEM
    - Nyquist plots.
    - Table of parameters
    - Snapshots of simulations
    - Plots of IL distribution
    - Leakage current
    - Conductance data

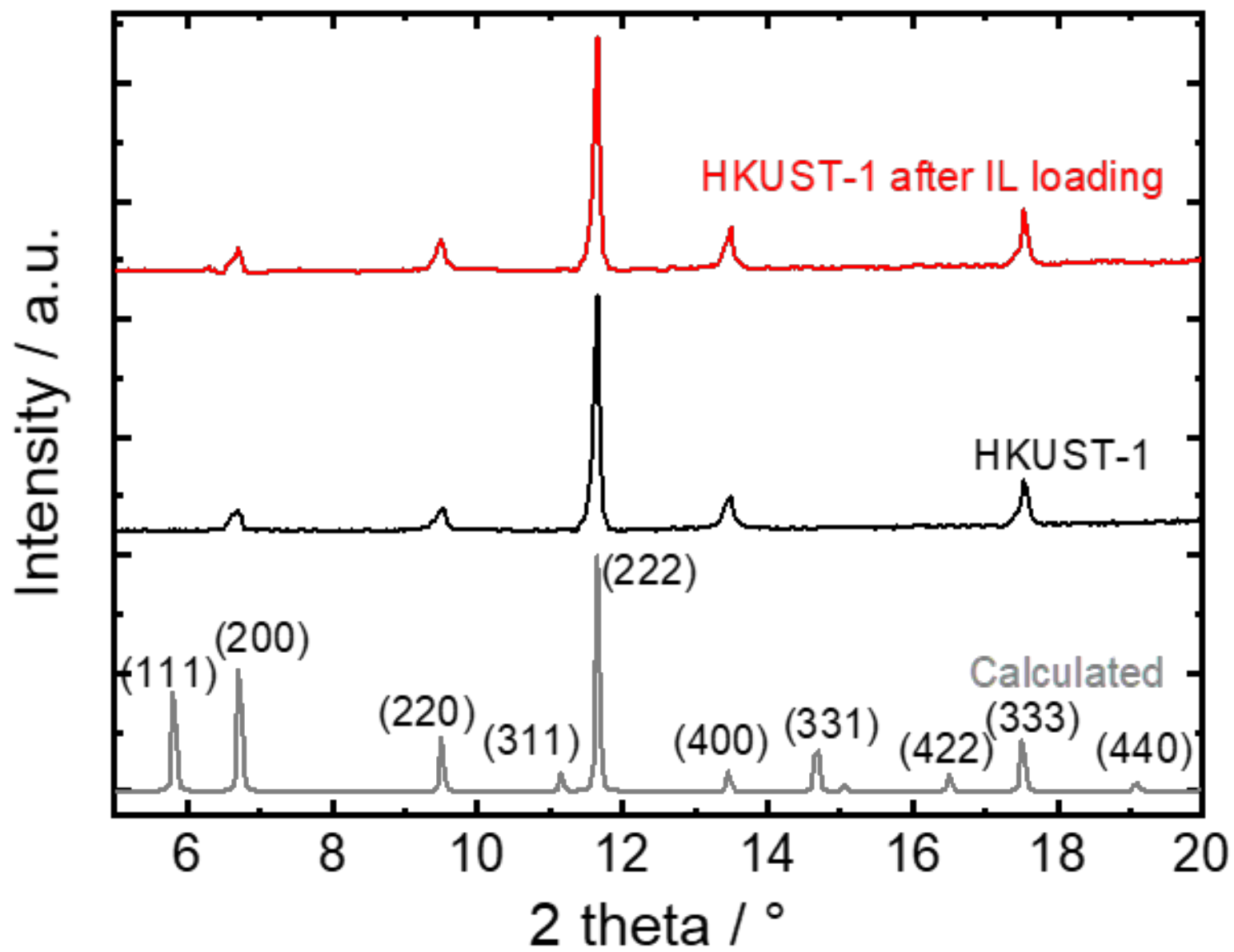


**Figure S1.** X-ray diffractograms (XRDs) of the HKUST-1 MOF films before (black) and after (red) IL loading. The observed diffraction peaks are labelled. The calculated XRD is shown in grey.

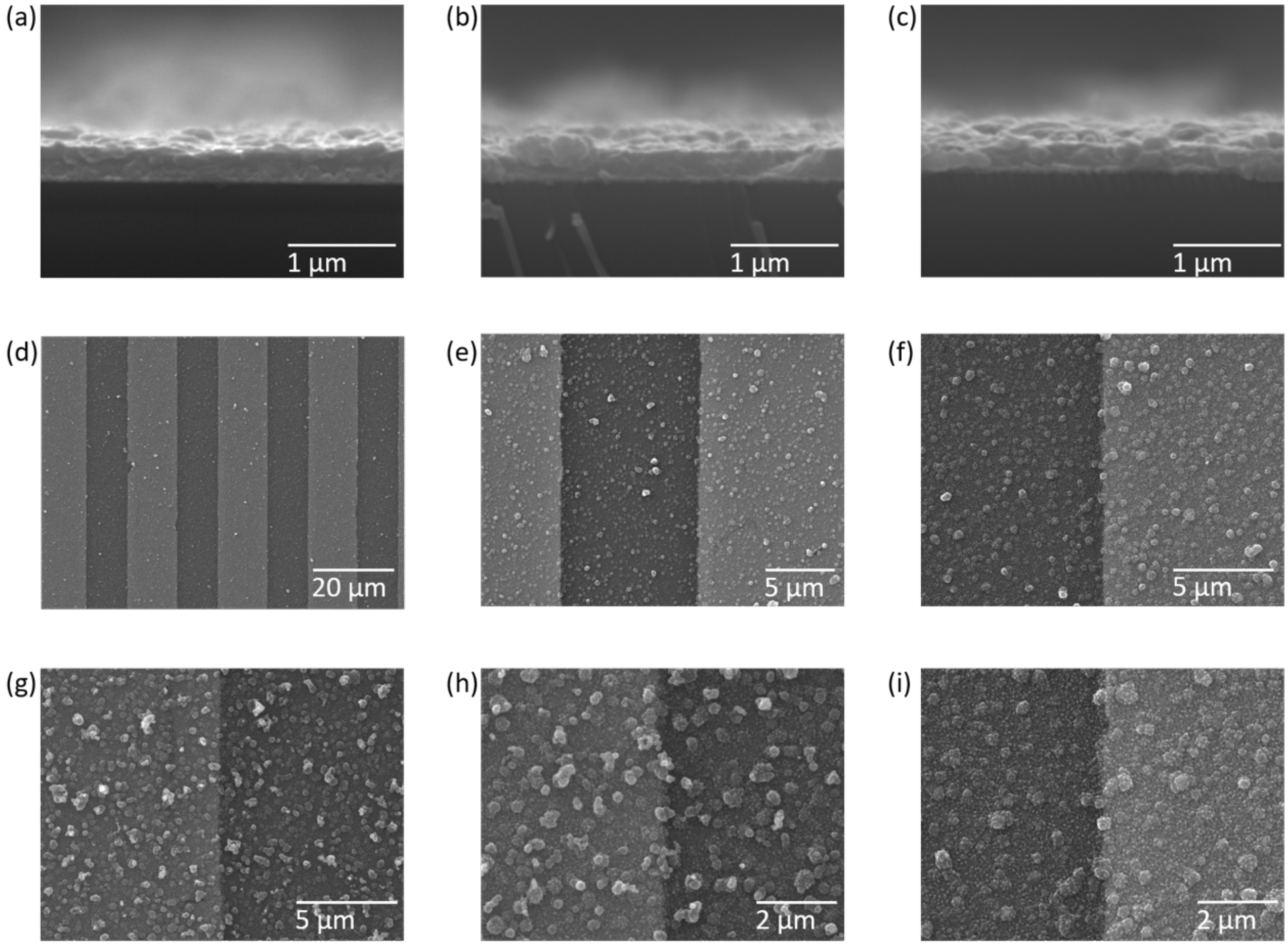


**Figure S2.** SEM images of cross-sections (a-c) and top views (d-i) of the HKUST-1 SURMOF film on the FET-substrate with the interdigitated electrodes. Panel e) shows a magnification of d). The top view image shows bright strips, which are the interdigitated gold electrodes on the substrate. The high-resolution images (g-i) show small HKUST-1 crystallites on top of the MOF film. The SURMOF film grew uniformly over the substrate (silicon oxide) and the gold electrodes. The estimated thickness of the SURMOF film is approximately 0.2 µm.

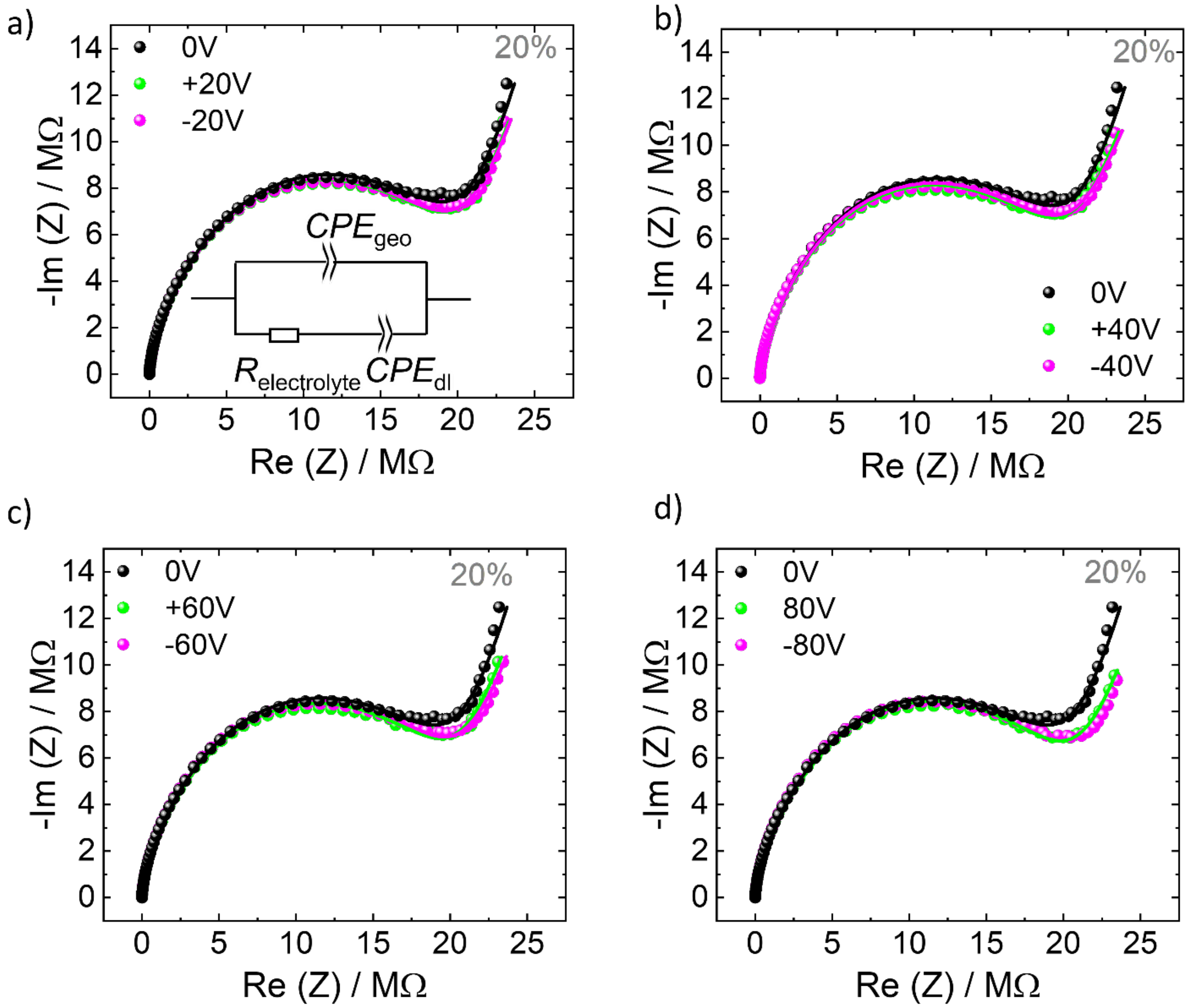


**Figure S3.** Nyquist plots of impedance data of the HKUST-1 sample with 20% IL pore filling at different gate voltages. The applied gate voltages are labeled. The frequency range is 5 MHz to 0.5 Hz. The impedance data were reproduced with the appropriate equivalent circuit, $(R\text{-}CPE_{dl})\|CPE_{geo}$, which is also shown in panel a). The spheres are the experimental data, the lines are the fits with the $(R\text{-}CPE_{dl})\|CPE_{geo}$ circuit. The parameters are given in Table S1.

.

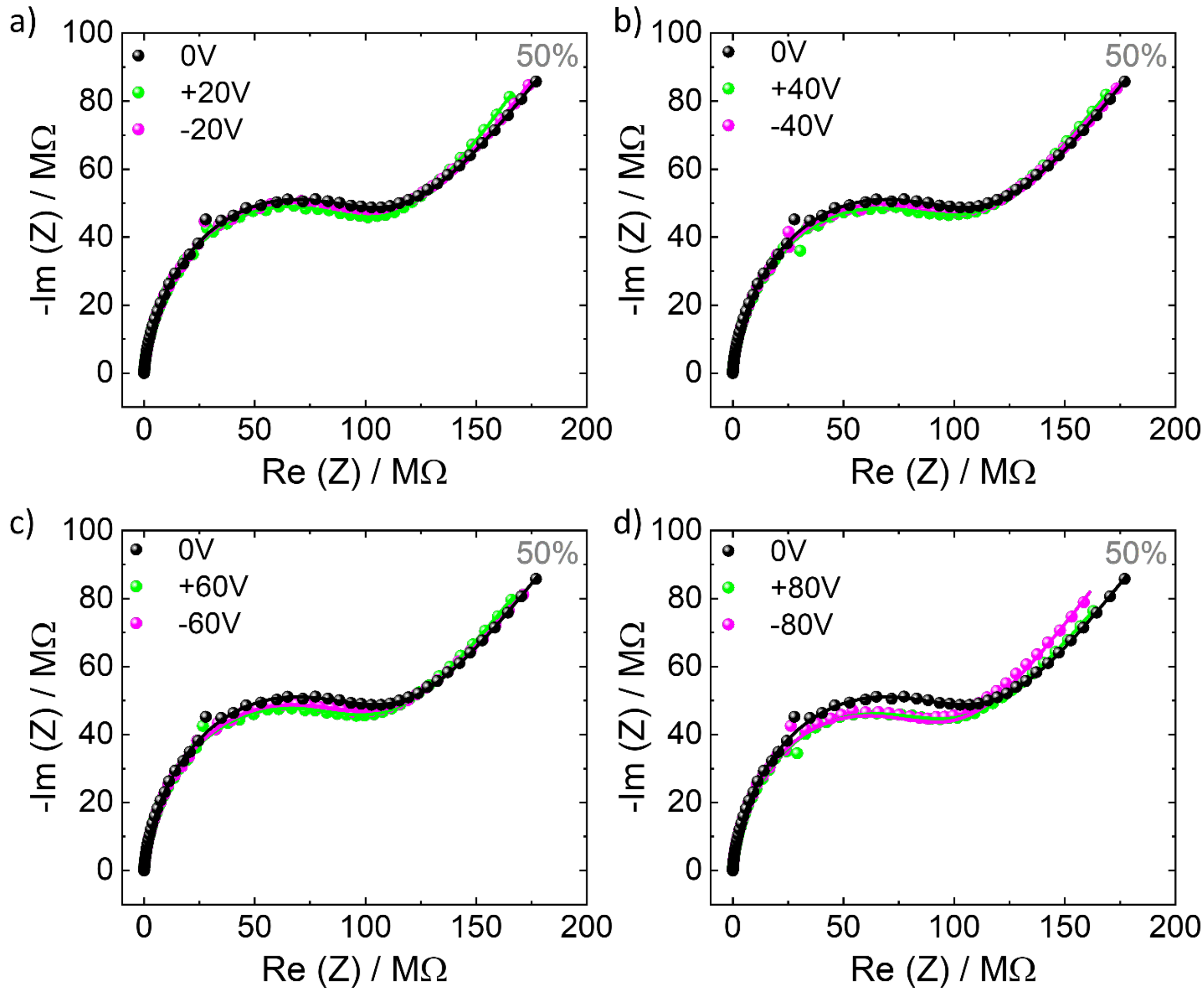


**Figure S4.** Nyquist plots of impedance data of the HKUST-1 sample with 50% IL pore filling at different gate voltages. The applied gate voltages are labeled. The frequency range is 5 MHz to 0.5 Hz. The impedance data were reproduced with the appropriate equivalent circuit, ($R$-$CPE_{dl}$)‖$CPE_{geo}$. The spheres are the experimental data, the lines are the fits with the ($R$-$CPE_{dl}$)‖$CPE_{geo}$ circuit. The parameters are given in Table S1.

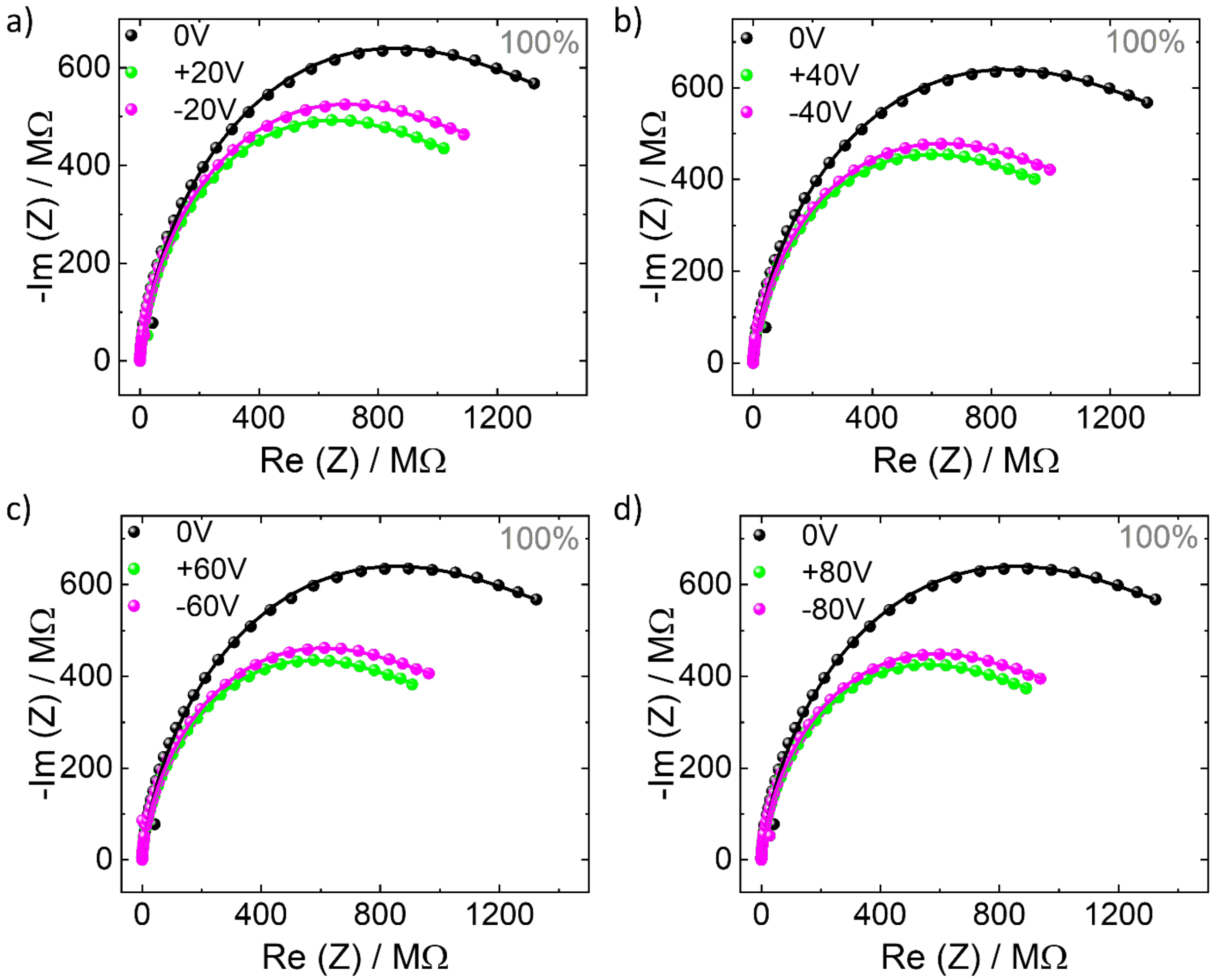


**Figure S5.** Nyquist plots of impedance data of the HKUST-1 sample with 100% IL pore filling at different gate voltages. The applied gate voltages are labeled. The frequency range is 5 MHz to 0.1 Hz. The impedance data were reproduced with the appropriate equivalent circuit, $(R\text{-}CPE_{dl})\|CPE_{geo}$. The spheres are the experimental data, the lines are the fits with the $(R\text{-}CPE_{dl})\|CPE_{geo}$ circuit. The parameters are given in Table S1.

The Nyquist plots at low loadings (Figure S3 and S4) show the characteristic semi-circle and low-frequency tail. Although the data at high loadings can also be described with the same equivalent circuit[4], these Nyquist plots only show the characteristic semi-circle and the low-frequency tail is not shown in the explored frequency range. (The semi-circle mainly represents the ohmic resistance of the system and the low-frequency tail mainly represents the double-layer-capacitance.[4]) Nevertheless, the data can be used to reliably determine the resistance and, thus, the conductivity.

**Table S1:** Parameters of the ($R$-$CPE_{dl}$)‖$CPE_{geo}$ circuit for describing the experimental data in Figures 2a-c, S3, S4 and S5. First 20% loading, then 50% and 100%.
The χ-value corresponds to the square-root of the mean-square-deviation between the experimental data and the model. The χ-values are usually in the order of 1% of the determined ohmic resistance or much smaller, indicating that the fits with the used model have only a small deviation from the experimental data.

**20% loading**

| Gate Voltage / V | $R$ / MΩ | $CPE_{dl}$ / $10^{-9}$ F $S^{n}dl^{-1}$ | $n_{dl}$ | $CPE$geo / $10^{-12}$ F $S^{n}geo^{-1}$ | $n_{geo}$ | χ / MΩ |
|---|---|---|---|---|---|---|
| 0 | 22.0512 | 10.6567 | 0.74604 | 101.767 | 0.83182 | 0.15167 |
| 20 | 21.1103 | 8.75566 | 0.74077 | 103.346 | 0.83237 | 0.14279 |
| -20 | 21.1589 | 8.76426 | 0.73826 | 101.506 | 0.83426 | 0.1398 |
| 0 | 21.4346 | 9.40008 | 0.73629 | 103.679 | 0.83043 | 0.15445 |
| 40 | 20.9387 | 9.44319 | 0.7281 | 103.484 | 0.83175 | 0.16422 |
| -40 | 20.8663 | 9.89607 | 0.71324 | 99.4599 | 0.83806 | 0.14193 |
| 0 | 21.3364 | 8.904 | 0.74145 | 104.501 | 0.83006 | 0.15222 |
| 60 | 21.286 | 9.34223 | 0.73985 | 106.66 | 0.82789 | 0.1592 |
| -60 | 21.414 | 9.5811 | 0.73213 | 99.3038 | 0.83565 | 0.13926 |
| 0 | 21.4522 | 7.56684 | 0.76816 | 107.278 | 0.82816 | 0.15345 |
| 80 | 21.5446 | 10.2579 | 0.73321 | 105.618 | 0.828 | 0.16077 |
| -80 | 21.65671 | 11.2794 | 0.71371 | 96.2337 | 0.83878 | 0.14566 |
| 0 | 21.5647 | 6.71744 | 0.76927 | 101.149 | 0.83506 | 0.14337 |
| 100 | 21.6701 | 12.3342 | 0.71352 | 96.577 | 0.83805 | 0.15675 |
| -100 | 21.8069 | 12.9062 | 0.73234 | 100.09 | 0.83391 | 0.12777 |
| 0 | 21.617 | 6.53238 | 0.77272 | 100.733 | 0.83517 | 0.16877 |

**50% loading**

| Gate Voltage / V | ***R*** **/ MΩ** | ***CPE*dl / $10^{-9}$ F $S^n$dl$^{-1}$** | ***n*dl** | ***CPE*geo / $10^{-12}$ F $S^n$geo$^{-1}$** | ***n*geo** | **χ / MΩ** |
|---|---|---|---|---|---|---|
| 0 | 103.684 | 9.97788 | 0.50638 | 98.4408 | 0.89607 | 0.49642 |
| 20 | 99.0363 | 10.4041 | 0.50647 | 99.7692 | 0.90047 | 0.49576 |
| -20 | 97.952 | 9.87982 | 0.48265 | 89.7269 | 0.90678 | 0.49548 |
| 0 | 99.7654 | 9.73564 | 0.47999 | 89.5442 | 0.90382 | 0.50496 |
| 40 | 95.6791 | 10.2063 | 0.48025 | 98.0292 | 0.89583 | 0.49126 |
| -40 | 96.2601 | 9.97858 | 0.47266 | 91.2444 | 0.9031 | 0.48465 |
| 0 | 101.021 | 9.49291 | 0.4698 | 88.8401 | 0.89802 | 0.52274 |
| 60 | 93.2409 | 10.4711 | 0.4751 | 97.4529 | 0.90025 | 0.49523 |
| -60 | 94.4887 | 10.219 | 0.46613 | 94.1019 | 0.89886 | 0.52935 |
| 0 | 97.3564 | 9.67114 | 0.45564 | 84.7549 | 0.90761 | 0.47307 |
| 80 | 88.6533 | 10.829 | 0.45529 | 100.0453 | 0.89514 | 0.45583 |
| -80 | 88.4737 | 10.2474 | 0.48802 | 92.938 | 0.90849 | 1.07817 |
| 0 | 99.3413 | 9.13337 | 0.46568 | 84.0003 | 0.90061 | 0.69038 |
| 100 | 83.6968 | 10.402 | 0.46486 | 89.2576 | 0.9171 | 0.55145 |
| -100 | 84.8 | 9.85981 | 0.45297 | 88.5501 | 0.91717 | 0.49543 |
| 0 | 97.133 | 9.00748 | 0.46654 | 81.0556 | 0.913 | 0.64587 |

**100% loading**

| Gate Voltage / V | *R* / MΩ | *CPE*dl / $10^{-9}$ F $S^{n_{dl}-1}$ | $n_{dl}$ | *CPE*geo / $10^{-12}$ F $S^{n_{geo}-1}$ | $n_{geo}$ | χ / MΩ |
|---|---|---|---|---|---|---|
| 0 | 1143.3042 | 2.69669 | 0.31258 | 67.11 | 0.89685 | 4.06018 |
| 20 | 887.3028 | 3.48421 | 0.3346 | 81.73 | 0.90476 | 2.63017 |
| -20 | 945.0738 | 3.21423 | 0.34428 | 75.55 | 0.9076 | 2.8007 |
| 0 | 1164.4884 | 2.70203 | 0.31974 | 66.24 | 0.89839 | 4.66999 |
| 40 | 818.0136 | 3.70424 | 0.34075 | 87.27 | 0.90558 | 2.86691 |
| -40 | 862.1298 | 3.51934 | 0.34121 | 82.40 | 0.90568 | 3.12862 |
| 0 | 1163.2824 | 2.68369 | 0.33152 | 64.99 | 0.9019 | 3.80956 |
| 60 | 784.0134 | 3.87683 | 0.34119 | 90.21 | 0.90621 | 2.87191 |
| -60 | 831.4974 | 3.66241 | 0.34237 | 84.45 | 0.90767 | 3.19601 |
| 0 | 1154.0718 | 2.6737 | 0.33586 | 64.20 | 0.90452 | 4.35335 |
| 80 | 767.1492 | 3.96426 | 0.34264 | 90.86 | 0.90786 | 2.37221 |
| -80 | 806.8284 | 3.71637 | 0.3467 | 85.10 | 0.91012 | 2.52748 |
| 0 | 1145.9052 | 2.67485 | 0.33609 | 64.08 | 0.90385 | 3.54822 |
| 100 | 749.1402 | 4.0147 | 0.33751 | 92.57 | 0.90626 | 2.56846 |
| 0 | 1140.4476 | 2.68265 | 0.33931 | 63.52 | 0.90657 | 3.66054 |
| -100 | 780.7248 | 3.87156 | 0.34317 | 87.23 | 0.91068 | 2.30389 |

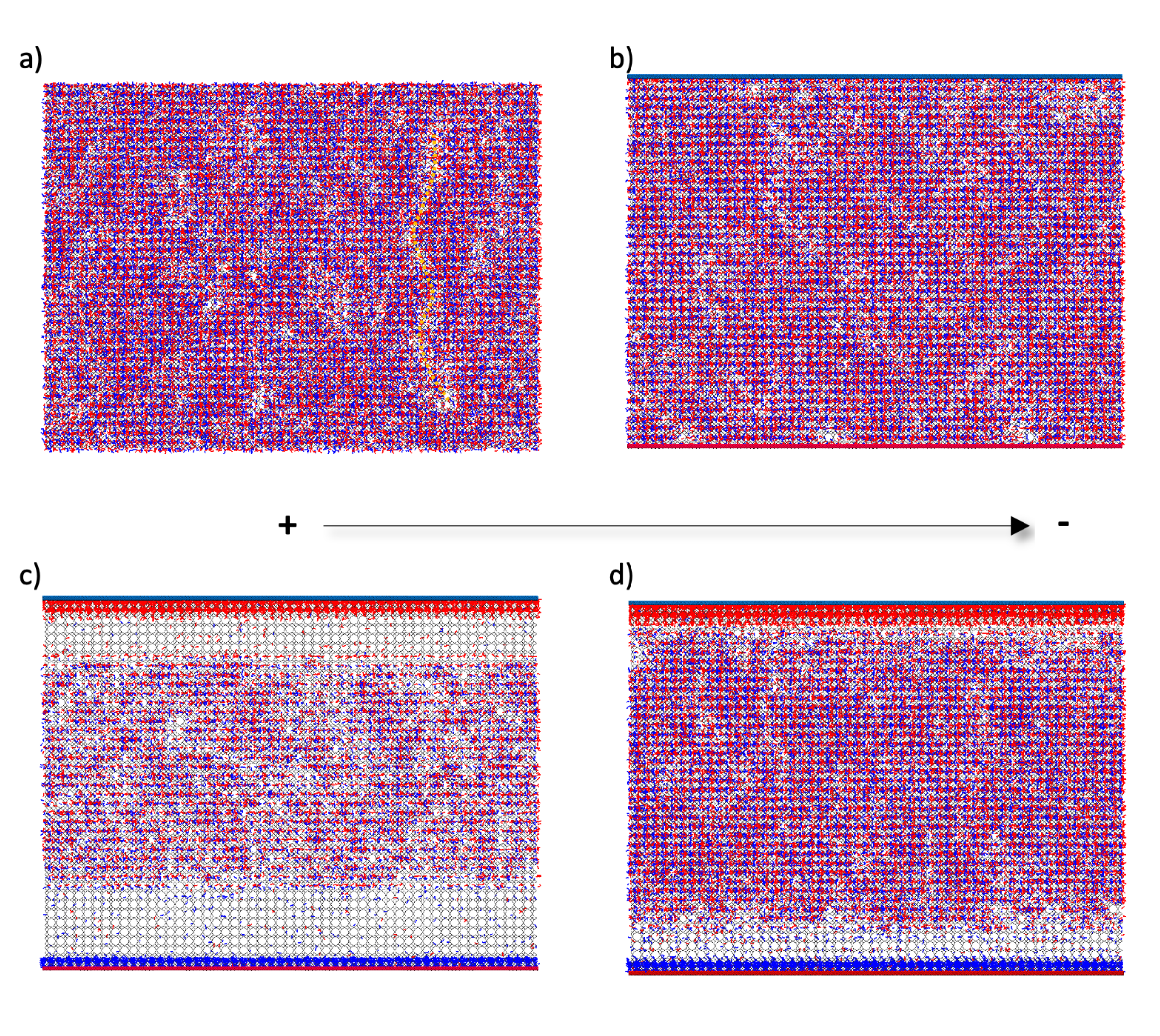


**Figure S6.** Snapshots of coarse-grained simulation with wrapped coordinates under periodic boundary conditions (PBC) in the xy-plane for: **a)** "bulk phase" IL@HKUST-1 at 100% IL loading and *z*-direction PBC, **b)** gated IL@HKUST-1 at 100% IL loading with zero bias in the gate electrodes, **c)** gated IL@HKUST-1 at 50% IL loading with ±100 V bias, and **d)** gated IL@HKUST-1 at 100% IL loading with ±100 V bias. The color-coded components correspond to HKUST-1 with 27x3x20 unitcells in gray, BMIM in red, TFSI in blue. For gated cases, the cathodes are marked in dark red and anodes in dark blue colors. In all scenarios, an *x*-direction electric field (~700 V) is applied and (partial) IL bunching is observed. For 100% IL loading, the bunching layers with highly concentrated IL ions leave low density "white cracks", a more prominent layered structure is indicated by the orange dashed line for the periodic IL@HKUST-1 in a). In the low IL loading case in c), the "white cracks" (i.e. area with a very small local ion density) are highly mobile, see **Movie S1**.

Under the 2D electric field, two types of double layer structures are formed: in the "bulk phase" IL@MOF due to the bunching effect, and the Helmholtz double layers at the MOF-electrolyte interface. According to the experiment, the *x*-direction electric field corresponds to an AC current, whilst the gate electrode along *z*-direction is charged with DC current. In a short time-scale in the "bulk phase" IL@MOF, by combining the two electric fields, mutual hinderance of cations and anions takes place in both directions, compressing the IL along both channel axes towards the critical IL density for bunching.

In the high-IL-concentration layers (with the mutual pore blocking) induced by 1D electric field, in a periodic system, a two-humps distribution of cations and anions with a bi-ionic high-concentration region in between can be observed. Particularly, since [BMIM] is more mobile than [TFSI], these IL-bunching layer slowly moves along the *E*-field, as the "group speed" of the ions rather than the individual speed of ions decides the overall IL mobility. By adding the

second *E*-field in the *z*-direction where the periodic boundary condition is broken by the explicit electrodes, the compression in the "bulk phase" in both the *x*- and *z*-directions remain comparable, however the interfacial double layers are also formed on the electrodes. In the "bulk phase", the maximum packing cannot be exceeded compared to the 1D *E*-field scenario, such that the second compression enforces a two-humps (double layer) distribution with less spatial offset, in **Figure S8**. Together with the ion accumulation within the interfacial double layers, low density ion channels are created to feature the aforementioned high IL mobility. With variable applied electric fields, the thickness, the in-layer IL density, and the IL mobility can be tuned.

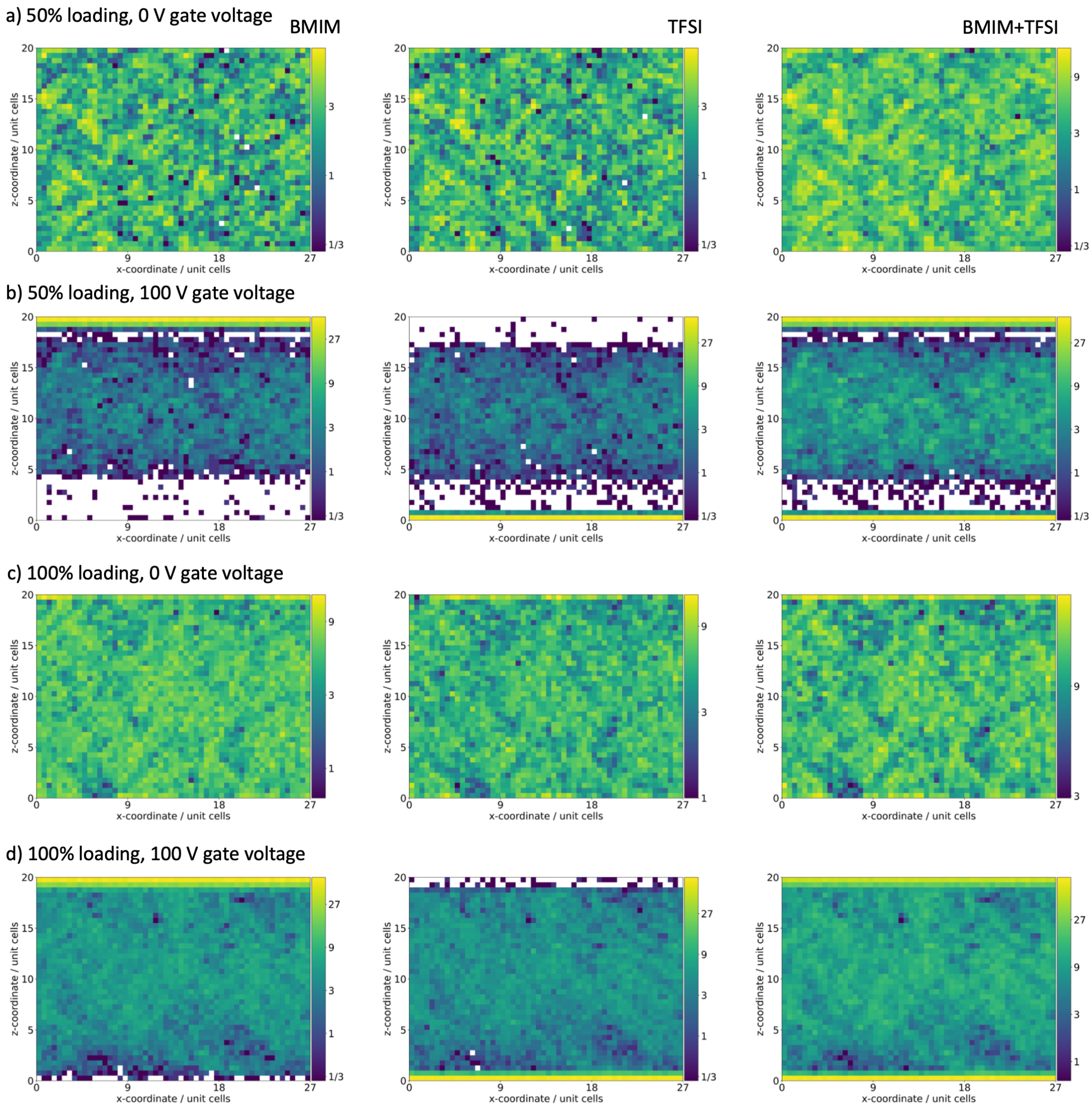


**Figure S7.** Local IL densities in the xz-cross-section for the snapshots of IL@HKUST-1 corresponding to 50% (a and b) *vs*. 100% (c and d) IL loading, and non-biased (a and c) vs. biased (b and d) scenarios in Figure S6. Among all four scenarios, the density of BMIM is shown in the first column, TFSI the second column, the combined BMIM+TFSI in the third column. Bunching layers are identified in c), third column as bright colored, interconnected MOF pores. In the biased scenario b) and d), the gate voltage in the *z*-direction creates a wide low density ion channels for 50% IL loading and a narrow channel in 100% IL loading. In particular in b), the ion channels feature virtually mono-species ion flow, with hardly any counter ion. The color scales correspond to local ion density, in units of ion per u.c. for cations or anions, and IL pairs per u.c. for BMIM+TFSI.

a) 15% IL loading

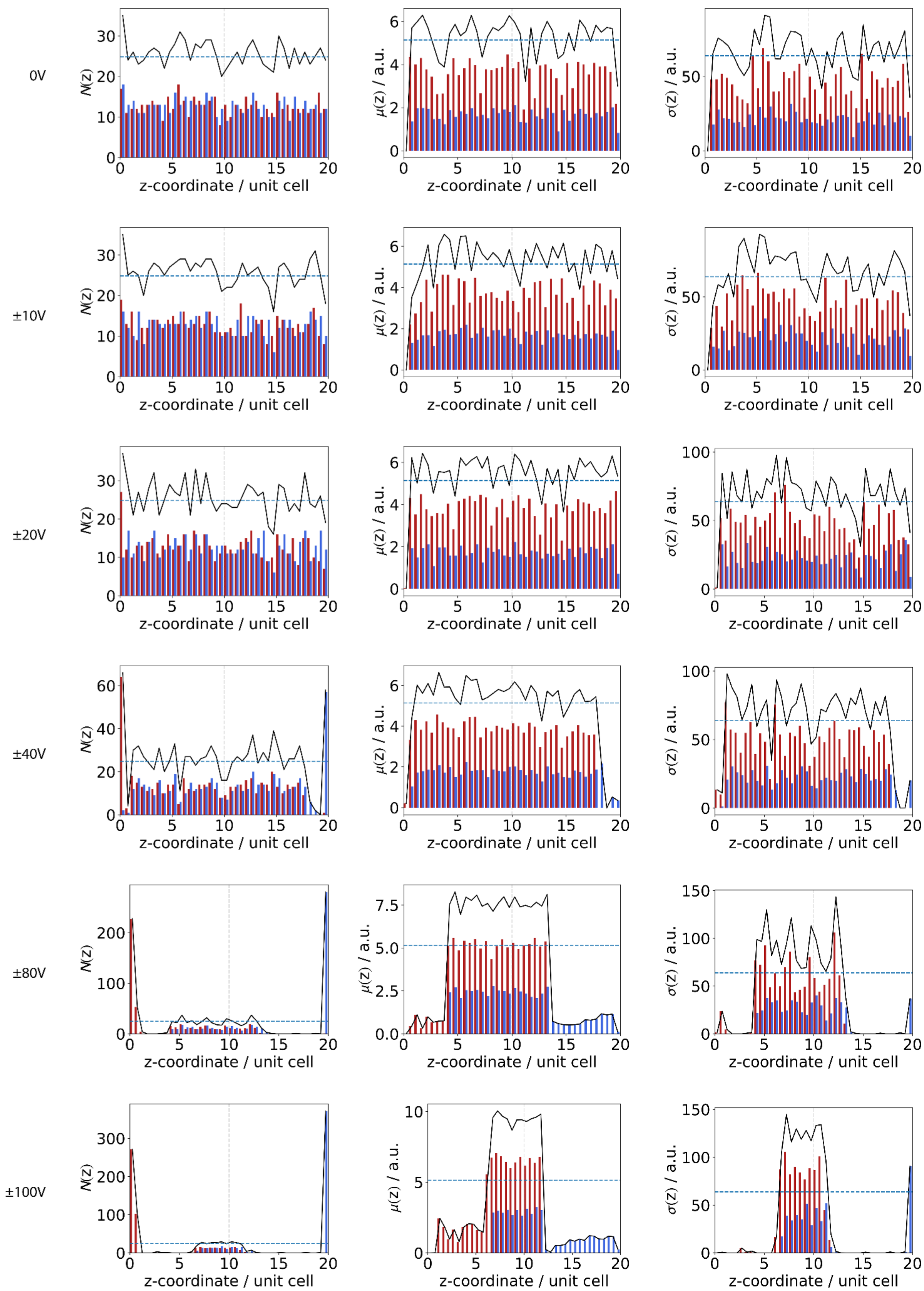

b) 30% IL loading

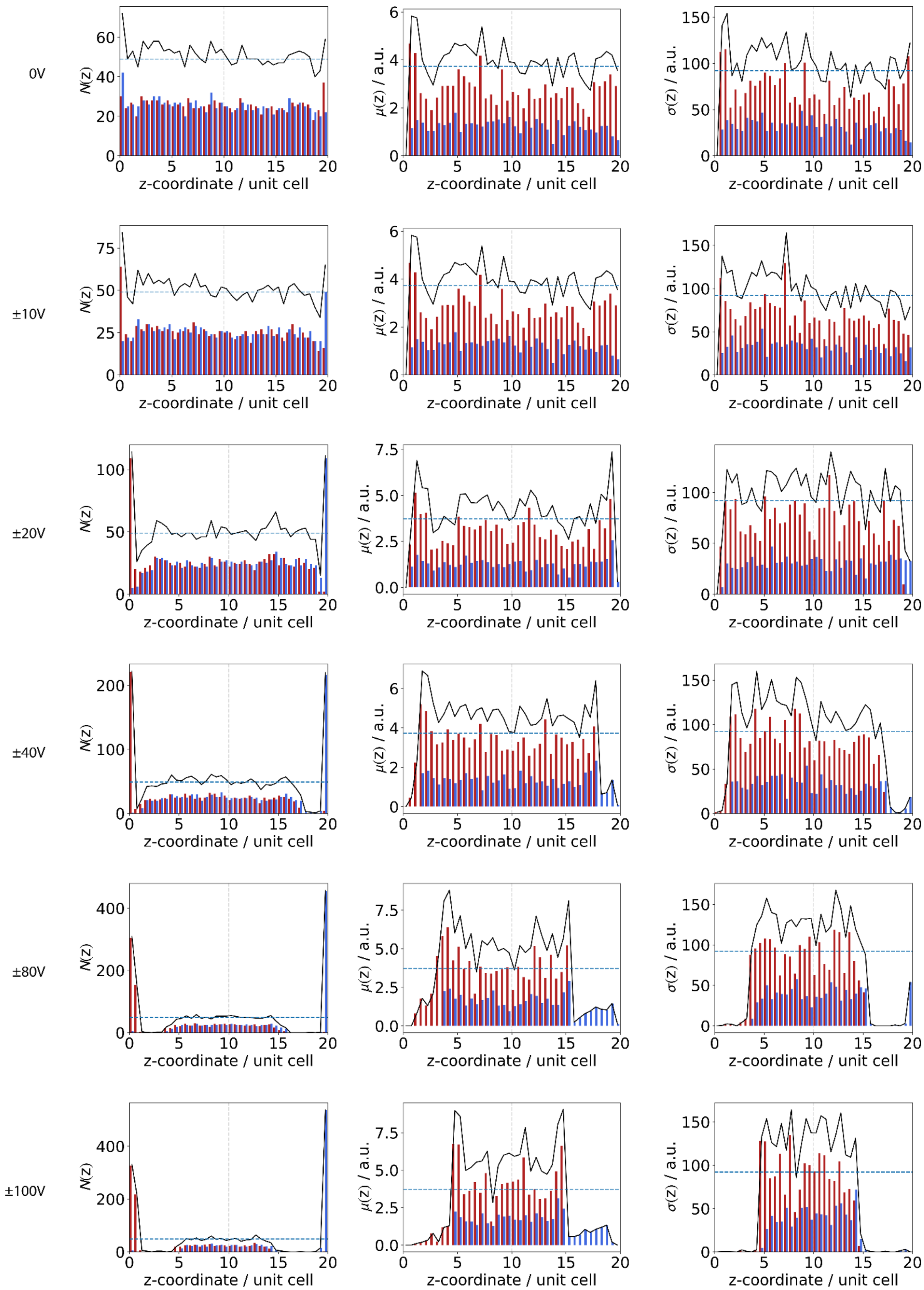

c) 50% IL loading

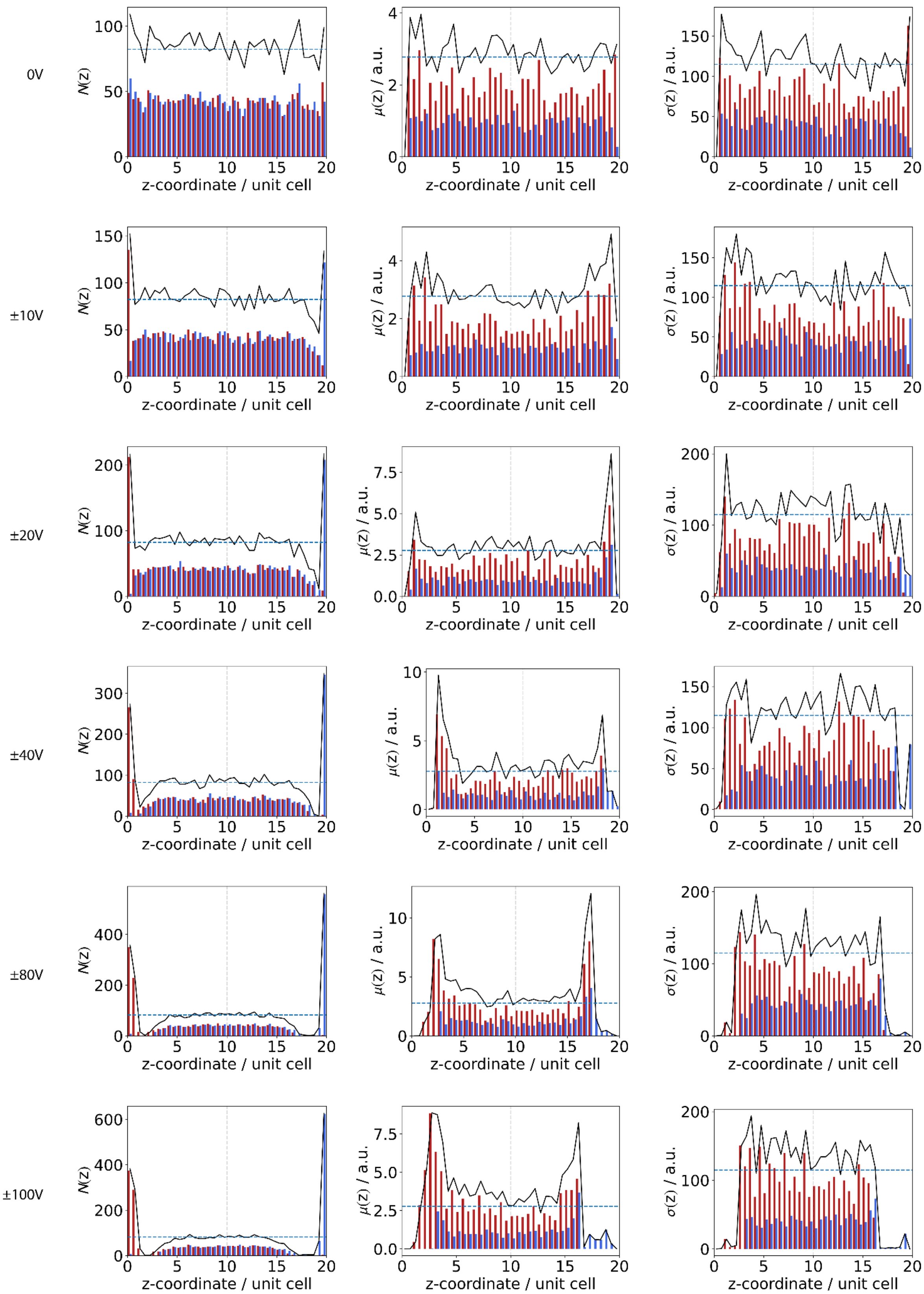

d) 70% IL loading

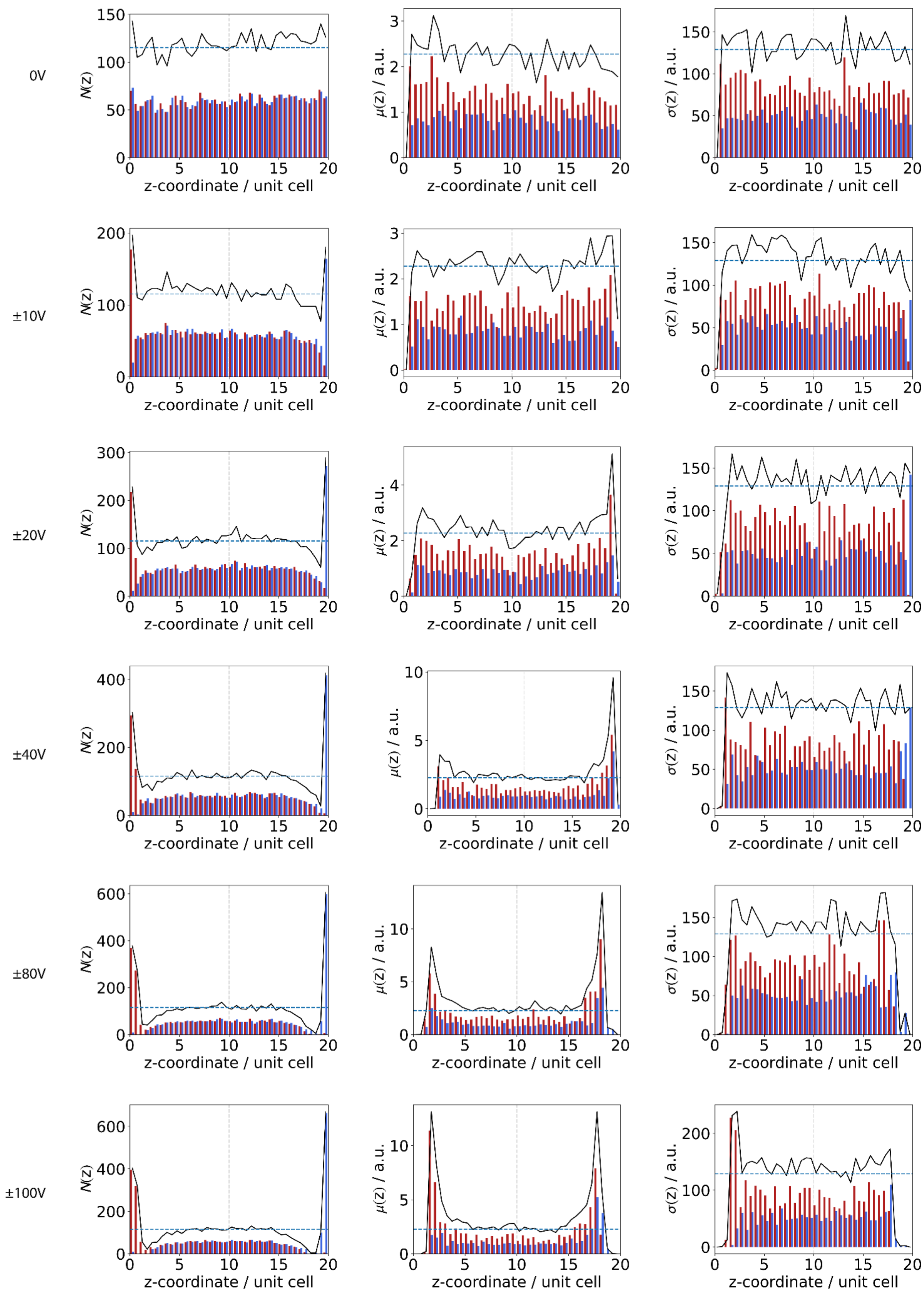

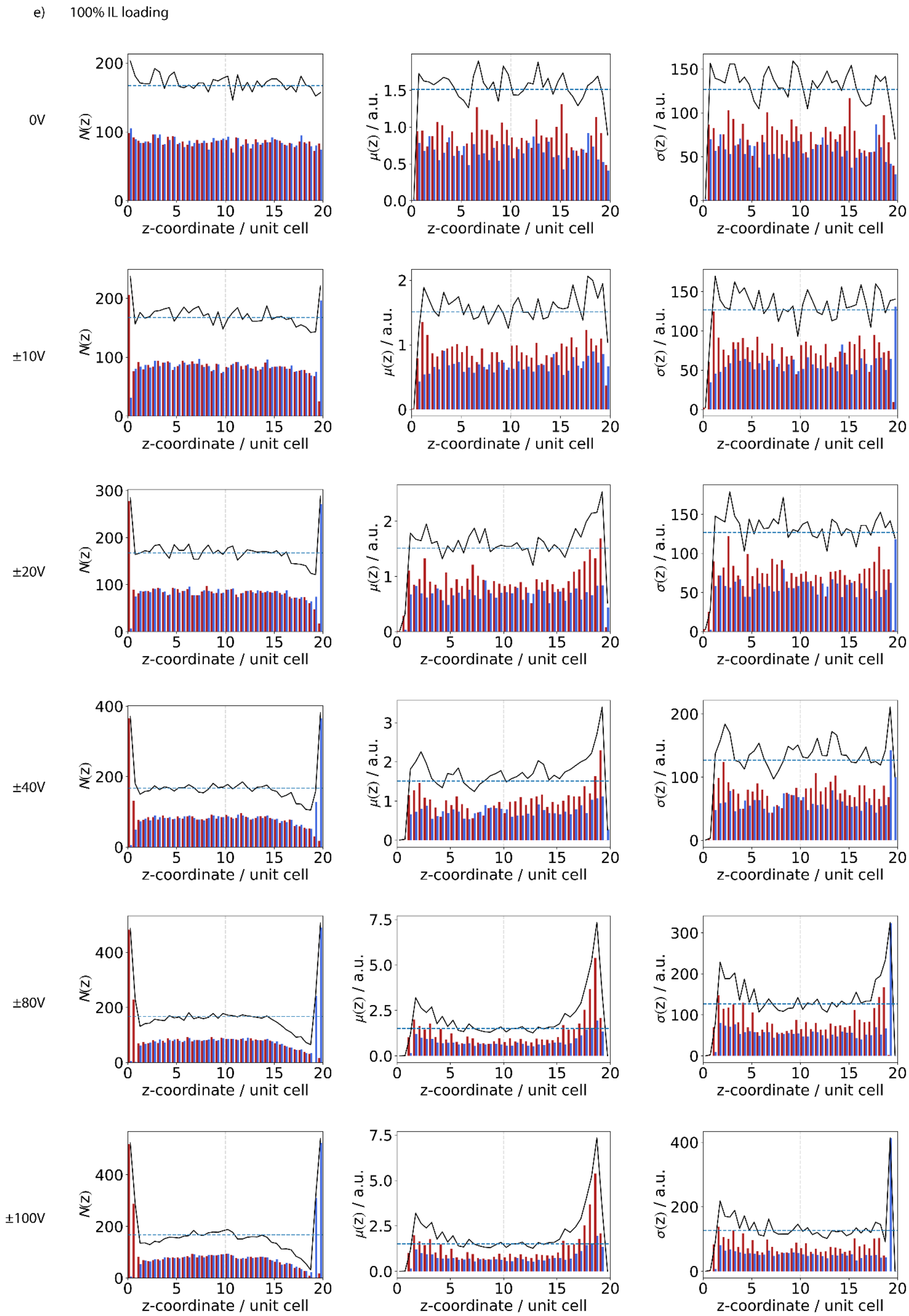


**Figure S8.** IL loading dependent Z-distribution of number density, mobility and conductivity for a) 15%, b) 30%, c) 50%, d) 70%, and e) 100% IL loadings under variable bias gate voltages. In the double plate setup, the explicit electrodes are charged to $\pm V_0$ for $V_0 \in 10\,\mathrm{V}, 20\,\mathrm{V}, 40\,\mathrm{V}, 80\,\mathrm{V}, 100\,\mathrm{V}$. Due to asymmetric cavities in the MOF-electrode interfaces half of the data are discarded ($z$ from 0-10 unit cells) during the calculation of average mobility increase in **Table S2** and conductivity in **Table S3**.

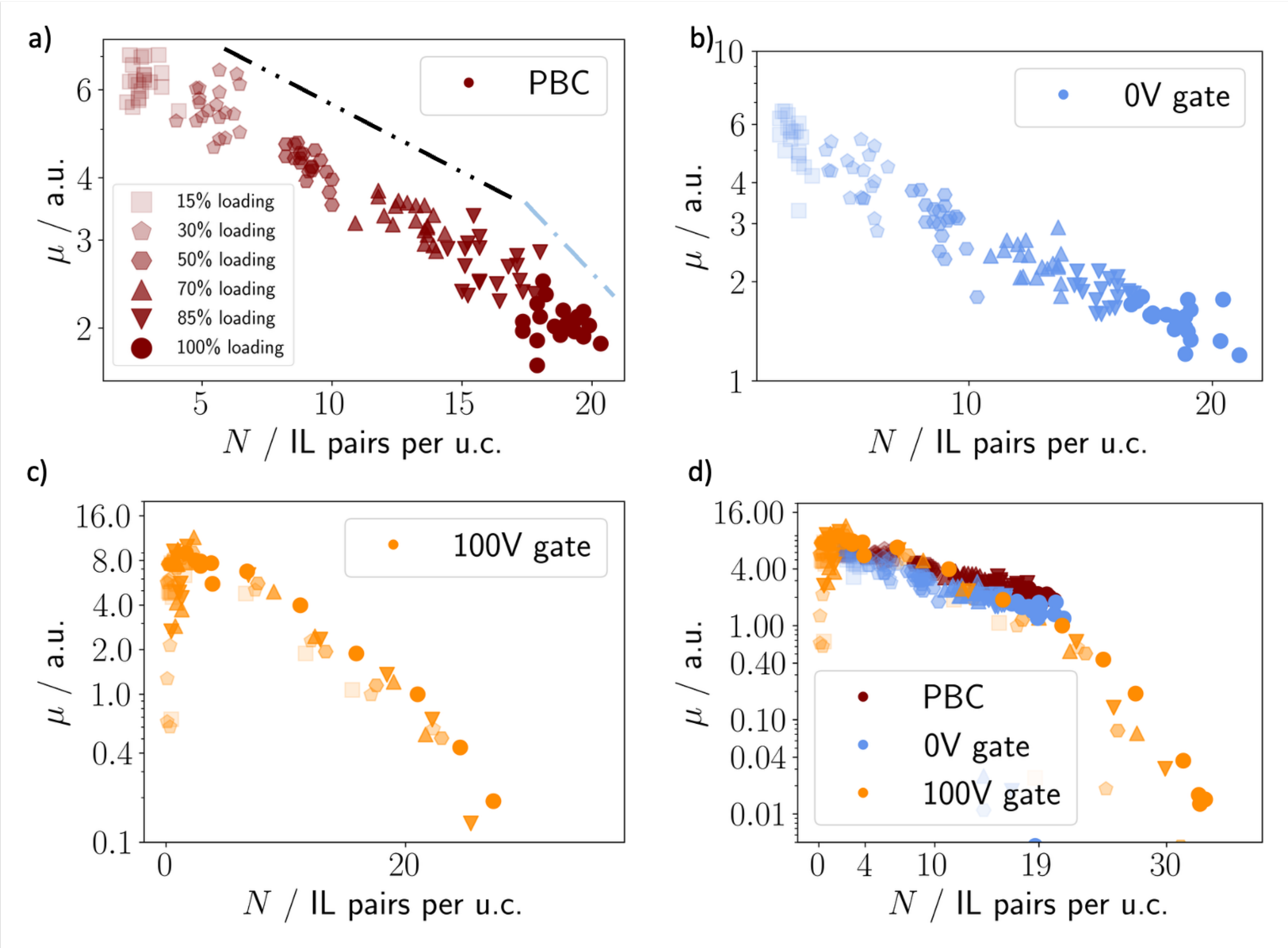


**Figure S9.** Local IL mobility *vs*. density for **a)** bulk phase IL@HKUST-1 (periodic boundary conditions, PBC) **b)** 0 V gate voltage and **c)** 100 V gate voltage under various IL loadings. When overlapped, all data points fall on an identical IL density-vs-mobility curve in d). The local densities and mobilities are summarized from *z*-direction slices (individual histogram bars) in **Figure S8**, where each sampling slice is of ½ thickness of the HKUST pore channel. The occasional outlier data points correspond to ions immobilized in the MOF-electrode interface while all remaining data points fall on a master curve shown in **Figure 6**. In general, fluctuations of IL mobility and density increase with respect to increased IL loading whilst individual data points are found in local clusters in a) and b). When the gate voltage is applied in c), for each IL loading, the local IL densities span across the entire range from low density ion channels to highly stacked in MOF-electrode interfaces. The bias voltage causes an IL distribution where in the ion channels close to the MOF-electrode interfaces are formed, where virtually only cations or anions remain. In the low IL loading case, a mono-species ion transport is ≈30% faster than in the non-biased cases. However, this mobility enhancement is quickly lost as soon as cations and anions are mixed. For medium to high loading, all curves overlap. For extremely high loadings, even if all ions travel in the same direction, the high local density also induces pore-blockage by the ions causing IL ion bunching.

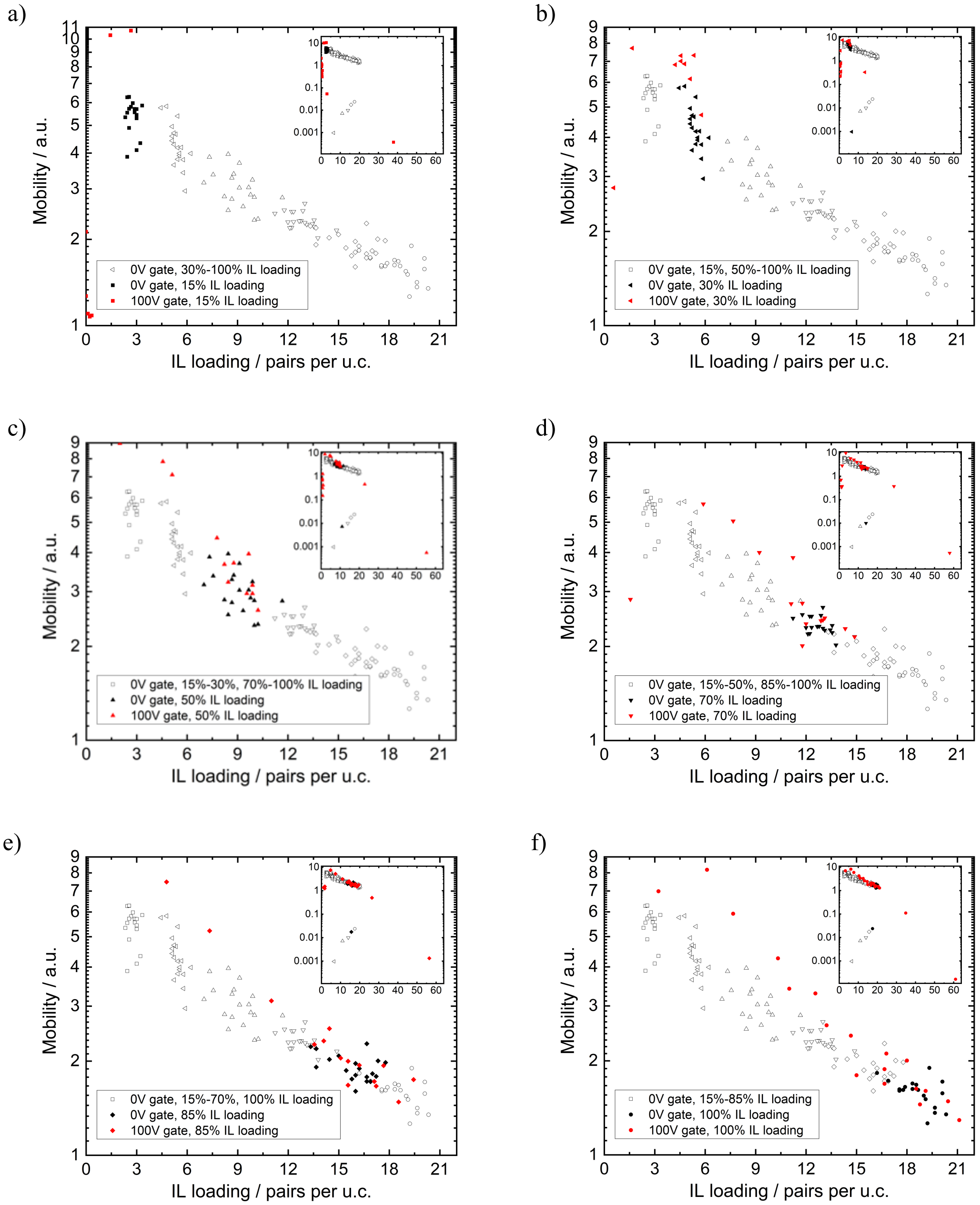


**Figure S10**. Local mobility *vs.* density coupling between 0-bias and biased scenarios with highlighted $N(z)$, $\mu(z)$ coupling under a) 15% IL loading, b) 30% IL loading, c) 50% IL loading, d) 70% IL loading, e) 85% IL loading, and f) 100% IL loading. The local densities and mobilities are summarized from *z*-direction slices (individual histogram bars) in **Figure S8**, where each sampling slice is of ½ thickness of the HKUST pore channel. The occasional outlier data points correspond to ions immobilized in the MOF-electrode interface while all remaining data points fall on a master curve shown in **Figure 6**. Tight groups of black hollow symbols correspond to 0-bias cases whereas under 100 V bias, the red data points disperse across the $(N, \mu)$ phase space with a consistent scaling as in Figure S9, to feature areas of high density ion bunching as well as low density ion highways. For each specific IL loading, except for very few immobilized ions in the secondary pores in the HKUST-1, the red data points under the biased case is always higher than the black ones for low loading and the two converge for high loading / in the "bulk phase". In the low-density-ion-highway, ion mobility is boosted due to a highly biased ion distribution (cation-rich near the anode and anion-rich near the cathode) resulting in a reduced cation-anion hinderance in the ion flow as well as Coulombic repellence among co-ions causing a more homogenous distribution.

**Table S2.** Average increase of IL mobility (μ) when a bias gate voltage is applied with respect to the 0-bias gate voltage mobility. The data were averaged for the different *z*-layers, Figure S8, which contain different amount of ions. The data were determined within an interval of $100\tau_{CG}$. A bias voltage of 100 V corresponds to a field strength of 3.9 V/nm. This is generally comparable to the electric field strength in *x*-direction with periodic boundary conditions (10 V/nm).

| IL loading | bias 10 V | bias 20 V | bias 40 V | bias 80 V | bias 100 V |
|---|---|---|---|---|---|
| 15% | 1.28% | 5.30% | 8.62% | 15.72% | 28.67% |
| 30% | 1.71% | 12.58% | 10.51% | 17.46% | 30.84% |
| 50% | 5.72% | 12.95% | 12.94% | 19.39% | 32.73% |
| 70% | 1.87% | 6.41% | 16.10% | 31.28% | 44.42% |
| 100% | 2.40% | 4.89% | 9.14% | 22.45% | 34.29% |

**Table S3.** Average increase of IL conductivity (σ) when a bias gate voltage is applied with respect to the 0-bias gate voltage conductivity. (The conductivity is averaged over all ions.)

| IL loading | bias 10 V | bias 20 V | bias 40 V | bias 80 V | bias 100 V |
|---|---|---|---|---|---|
| 15% | 3.75% | 6.81% | -0.94% | -30.07% | -46.69% |
| 30% | 6.08% | 7.78% | 3.15% | -15.66% | -26.45% |
| 50% | 3.33% | 0.93% | -0.60% | -10.33% | -11.73% |
| 70% | 2.50% | 2.35% | 1.28% | -2.76% | -7.44% |
| 100% | 3.20% | 2.18% | 4.17% | 7.39% | 9.74% |

**Table S4.** Coarse-grained force-field parameters for individual CG components

| CG component | Mass | Charge | LJ-epsilon | LJ-sigma |
|---|---|---|---|---|
| MOF-SBU | 228.84 | 0.0 | 1.10 | 7.56 |
| MOF-linker | 75.05 | 0.0 | 0.90 | 4.32 |
| MOF dummy-secondary pore | 300.0 | 0.0 | 1.0 | 6.0 |
| Electrode-gold | 196.97 | Charge managed by automatic algorithm for electrodes | 0.80 | 2.0 |
| BMIM-head (-CH3) | 15.04 | 0.1578 | 0.086 | 3.8192 |
| BMIM-imidazole | 67.07 | 0.4374 | 0.612 | 4.9056 |
| BMIM-tail (C4) | 57.12 | 0.1848 | 0.437 | 5.6448 |
| TFSI-nexus | 142.13 | -0.5800 | 0.90 | 4.480 |
| TFSI-endgroups(CF3) | 69.01 | -0.1000 | 0.60 | 3.584 |

.

**Movie S1**. Animations of IL transport in HKUST-1 in coarse-grained molecular dynamics simulation.

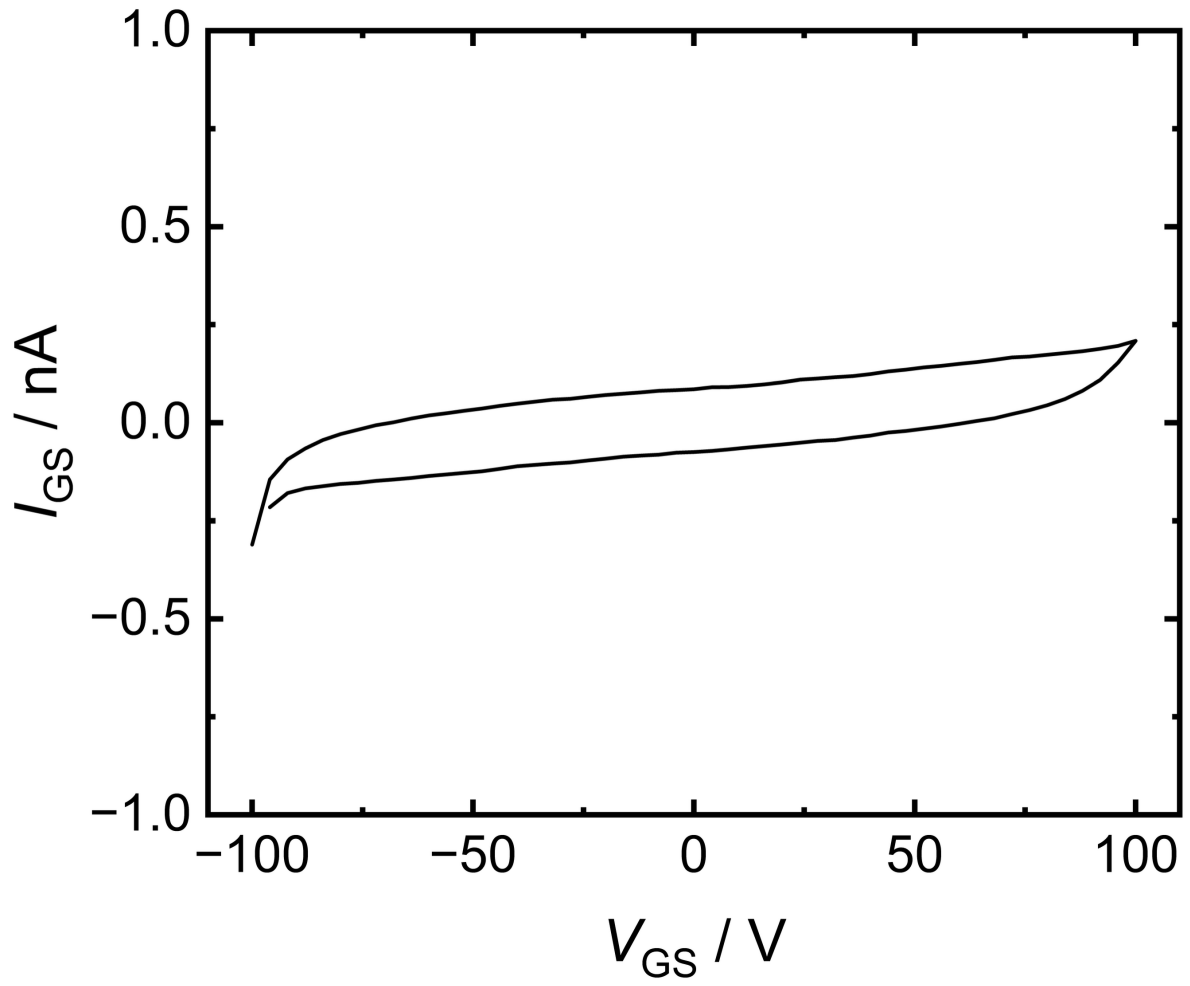


**Figure S11.** The leakage-current characteristics ($I_{GS}$ *vs*. $V_{GS}$). The leakage current ($I_{GS}$) remains below 1 nA during gate-voltage ($V_{GS}$) sweeping from -100 V to +100 V.

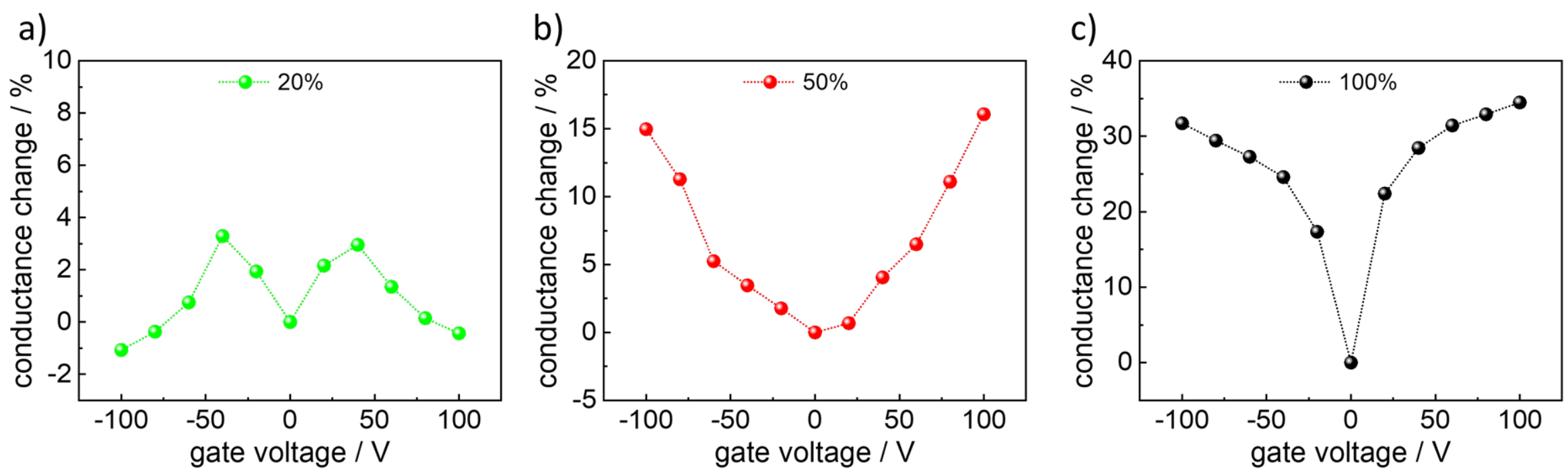


**Figure S12.** Conductance change as a function of the gate voltage. The IL pore filling is 20% (a), 50% (b) and 100% (c). The reference conductance values are obtained at a gate voltage of 0 V.
